# Supplementary material for: The derlin Dfm1 couples retrotranslocation of a folded protein domain to its proteasomal degradation
Source: J Cell Biol. 2024 Mar 5;223(5):e202308074. doi: 10.1083/jcb.202308074 (PMC11066878; doi:10.1083/jcb.202308074)
Supplement: Table S1 — lists yeast strains used in this study. [file JCB_202308074_TableS1.docx]

**Table S1. List of yeast strains used in this study**

| **Strain** | **Genotype** | **Reference** |
| --- | --- | --- |
| yPC1507 | Mata FY251 *ura3-52 his3D200 leu2D1 trp1D62* |  |
| yPC11111 | Mata *ura3-52 his3D200 leu2D1 trp1D81* ASI1::NAT URA3::GPDp-*At*Tir1-9myc HO::CPYp-3xHA-DHFR-OST(TM)-4aid-9myc::HYGR | This study |
| yPC11120 | Mata *ura3-52 his3D200 leu2D1 trp1D81* DOA10::HIS3 URA3::GPDp-*At*Tir1-9myc HO::CPYp-3xHA-DHFR-OST(TM)-4aid-9myc::HYGR | This study |
| yPC11124 | Mata *ura3-52 his3D200 leu2D1 trp1D81 hrd1Δ*(CRISPR) URA3::GPDp-*At*Tir1-9myc HO::CPYp-3xHA-DHFR-OST(TM)-4aid-9myc::HYGR | This study |
| yPC11143 | Mata *ura3-52 his3D200 leu2D1 trp1D81* UBC7::KAN URA3::GPDp-*At*Tir1-9myc HO::CPYp-3xHA-DHFR-OST(TM)-4aid-9myc::HYGR | This study |
| yPC11149 | Mata *ura3-52 his3D200 leu2D1 trp1D81* HO::CPYp-3xHA-DHFR-OST(TM)-4aid-9myc::HYGR | This study |
| yPC11151 | Mata *ura3-52 his3D200 leu2D1 trp1D81* RAD23::HIS DSK2::KAN URA3::GPDp-*At*Tir1-9myc HO::ADHp-3xHA-DHFR-OST(TM)-4aid-9myc::HYGR | This study |
| yPC11155 | Mata *ura3-52 his3D200 leu2D1 trp1D81* UBX2::KAN URA3::GPDp-*At*Tir1-9myc HO::ADHp-3xHA-DHFR-OST(TM)-4aid-9myc::HYGR | This study |
| yPC11156 | Mata *ura3-52 his3D200 leu2D1 trp1D81* UFD2::KAN URA3::GPDp-*At*Tir1-9myc HO::ADHp-3xHA-DHFR-OST(TM)-4aid-9myc::HYGR | This study |
| yPC11233 | Mata *ura3-52 his3D200 leu2D1 trp1D81* URA3::GPDp-*At*Tir1-9myc HO::CPYp-3xHA-DHFR-OST(TM)-4aid-9myc::HYGR | This study |
| yPC11234 | Mat? *ura3-52 his3D200 leu2D1 trp1D81* Asi1::NAT Doa10::HIS Hrd1::KAN URA3::GPDp-*At*Tir1-9myc HO::CPYp-3xHA-DHFR-OST(TM)-4aid-9myc::HYGR | This study |
| yPC11236 | Mata *ura3-52 his3D200 leu2D1 trp1D81* DER1::HIS3 URA3::GPDp-*At*Tir1-9myc HO::CPYp-3xHA-DHFR-OST(TM)-4aid-9myc::HYGR | This study |
| yPC11237 | Mata *ura3-52 his3D200 leu2D1 trp1D81* DFM1::KAN URA3::GPDp-*At*Tir1-9myc HO::CPYp-3xHA-DHFR-OST(TM)-4aid-9myc::HYGR | This study |
| yPC11238 | Mata *ura3-52 his3D200 leu2D1 trp1D81* DER1::HIS3 DFM1::KAN URA3::GPDp-*At*Tir1-9myc HO::CPYp-3xHA-DHFR-OST(TM)-4aid-9myc::HYGR | This study |
| yPC11263 | Mata *ura3-52 his3D200 leu2D1 trp1D81* RAD23::HIS DSK2::KAN DFM1::KAN URA3::GPDp-*At*Tir1-9myc HO::ADHp-3xHA-DHFR-OST(TM)-4aid-9myc::HYGR | This study |
| yPC11280 | Mat? *ura3-52 his3D200 leu2D1 trp1D81* Asi1::NAT Doa10::HIS Hrd1::KAN *dfm1Δ*(CRISPR) URA3::GPDp-*At*Tir1-9myc HO::CPYp-3xHA-DHFR-OST(TM)-4aid-9myc::HYGR | This study |
| yPC11282 | Mat? *ura3-52 his3D200 leu2D1 trp1D81* Asi1::NAT Doa10::HIS Hrd1::KAN *der1Δ*(CRISPR) URA3::GPDp-*At*Tir1-9myc HO::CPYp-3xHA-DHFR-OST(TM)-4aid-9myc::HYGR | This study |
| yPC11287 | Mata *ura3-52 his3D200 leu2D1 trp1D81* URA3::GPDp-*Os*Tir1-V5 HO::CPYp-3xHA-DHFR-OST(TM)-4aid-9myc::HYGR | This study |
| yPC11289 | Mat? *ura3-52 his3D200 leu2D1 trp1D81* *npl4-1* URA3::GPDp-*Os*Tir1-V5 HO::CPYp-3xHA-DHFR-OST(TM)-4aid-9myc::HYGR | This study |
| yPC11678 | Mat? *ura3-52 his3D200 leu2D1 trp1D81* *npl4-1* DFM1::KAN URA3::GPDp-*Os*Tir1-V5 HO::CPYp-3xHA-DHFR-OST(TM)-4aid-9myc::HYGR | This study |
| yPC11681 | Mata *ura3-52 his3D200 leu2D1 trp1D81* URA3::GPDp-*At*Tir1-9myc HO::ADHp-3xHA-DHFR-OST(TM)-4aid-9myc::HYGR | This study |
| yPC11682 | Mata *ura3-52 his3D200 leu2D1 trp1D81* DFM1::KAN URA3::GPDp-*At*Tir1-9myc HO::ADHp-3xHA-DHFR-OST(TM)-4aid-9myc::HYGR | This study |
| yPC11687 | Mata *ura3-52 his3D200 leu2D1 trp1D81* URA3::GPDp-*At*Tir1-9myc HO::ADHp-3xHA-yeGFP-DHFR-OST(TM)-4aid-9myc::HYGR | This study |
| yPC11688 | Mata *ura3-52 his3D200 leu2D1 trp1D81* DFM1::KAN URA3::GPDp-*At*Tir1-9myc HO::ADHp-3xHA-yeGFP-DHFR-OST(TM)-4aid-9myc::HYGR | This study |
| yPC12071 | Mata *ura3-52 his3D200 leu2D1 trp1D81* UFD2::KAN DFM1::HIS3 URA3::GPDp-*At*Tir1-9myc HO::ADHp-3xHA-DHFR-OST(TM)-4aid-9myc::HYGR | This study |
| yPC12074 | Mata *ura3-52 his3D200 leu2D1 trp1D81* URA3::GPDp-*At*Tir1-9myc HO::ADHp-3xHA-DHFR(29-32Pro)-OST(TM)-4aid-9myc::HYGR | This study |
| yPC12075 | Mata *ura3-52 his3D200 leu2D1 trp1D81* DFM1::KAN URA3::GPDp-*At*Tir1-9myc HO::ADHp-3xHA-DHFR(29-32Pro)-OST(TM)-4aid-9myc::HYGR | This study |
| yPC12076 | Mat? *ura3-52 his3D200 leu2D1 trp1D81* Asi1::NAT Doa10::HIS Hrd1::KAN URA3::GPDp-*At*Tir1-9myc HO::ADHp-3xHA-DHFR(29-31Pro)-OST(TM)-4aid-9myc::HYGR | This study |
| yPC12077 | Mat? *ura3-52 his3D200 leu2D1 trp1D81* Asi1::NAT Doa10::HIS Hrd1::KAN *dfm1Δ*(CRISPR) URA3::GPDp-*At*Tir1-9myc HO::ADHp-3xHA-DHFR(29-31Pro)-OST(TM)-4aid-9myc::HYGR | This study |
| yPC12081 | Mata *ura3-52 his3D200 leu2D1 trp1D81* URA3::GPDp-*At*Tir1 HO::ADHp-3xHA-DHFR-OST(TM)-4aid-9myc::HYGR | This study |
| yPC12152 | Mata *ura3-52 his3D200 leu2D1 trp1D81 pdr5Δ*(CRISPR) URA3::GPDp-*At*Tir1-9myc HO::ADHp-3xHA-DHFR-OST(TM)-4aid-9myc::HYGR | This study |
| yPC12153 | Mata *ura3-52 his3D200 leu2D1 trp1D81 pdr5Δ*(CRISPR) DFM1::KAN URA3::GPDp-*At*Tir1-9myc HO::ADHp-3xHA-DHFR-OST(TM)-4aid-9myc::HYGR | This study |
| YPC12207 | Mata *ura3-52 his3D200 leu2D1 trp1D81* DFM1::KAN PEP4::HIS3 URA3::GPDp-*At*Tir1-9myc HO::ADHp-3xHA-yeGFP-DHFR-OST(TM)-4aid-9myc::HYGR | This study |
| YPC12354 | Mata *ura3-52 his3D200 leu2D1 trp1D81* DFM1::KAN PEP4::HIS3 hrd*1Δ*(CRISPR) URA3::GPDp-*At*Tir1-9myc HO::ADHp-3xHA-yeGFP-DHFR-OST(TM)-4aid-9myc::HYGR | This study |
| yPC12355 | Mata *ura3-52 his3D200 leu2D1 trp1D81* UFD2::HIS3 URA3::GPDp-*At*Tir1-9myc HO::ADHp-3xHA-yeGFP-DHFR-OST(TM)-4aid-9myc::HYGR | This study |
| yPC12638 | Mat? *ura3-52 his3D200 leu2D1 trp1D81* URA3::GPDp-*Os*Tir1-V5 HO::ADHp-3xHA-DHFR-OST(TM)-4aid-9myc::HYGR | This study |
| yPC12639 | Mat? *ura3-52 his3D200 leu2D1 trp1D81* DFM1::KAN URA3::GPDp-*Os*Tir1-V5 HO::ADHp-3xHA-DHFR-OST(TM)-4aid-9myc::HYGR | This study |
| yPC12640 | Mat? *ura3-52 his3D200 leu2D1 trp1D81* *cdc48-6* URA3::GPDp-*Os*Tir1-V5 HO::ADHp-3xHA-DHFR-OST(TM)-4aid-9myc::HYGR | This study |
| yPC12641 | Mat? *ura3-52 his3D200 leu2D1 trp1D81* *cdc48-6* DFM1::KAN URA3::GPDp-*Os*Tir1-V5 HO::ADHp-3xHA-DHFR-OST(TM)-4aid-9myc::HYGR | This study |
| yPC12656 | Mata *ura3-52 his3D200 leu2D1 trp1D81* URA3::GPDp-*At*Tir1-9myc HO::ADHp-3xHA-DHFR-3V5-OST(TM)-4aid-9myc::HYGR | This study |
| yPC12657 | Mata *ura3-52 his3D200 leu2D1 trp1D81* DFM1::KAN URA3::GPDp-*At*Tir1-9myc HO::ADHp-3xHA-DHFR-3V5-OST(TM)-4aid-9myc::HYGR | This study |
| yPC12658 | Mata *ura3-52 his3D200 leu2D1 trp1D81* URA3::GPDp-*At*Tir1-9myc HO::ADHp-3xHA-DHFR-OST(TM)-3V5-4aid-9myc::HYGR | This study |
| yPC12659 | Mata *ura3-52 his3D200 leu2D1 trp1D81* DFM1::KAN URA3::GPDp-*At*Tir1-9myc HO::ADHp-3xHA-DHFR-OST(TM)-3V5-4aid-9myc::HYGR | This study |
| yPC12804 | Mata *ura3-52 his3D200 leu2D1 trp1D81* URA3::GPDp-*Os*Tir1-V5 HO::ADHp-3xHA-DHFR-3V5-OST(TM)-4aid-9myc::HYGR | This study |
| yPC12805 | Mata *ura3-52 his3D200 leu2D1 trp1D81* *dfm1Δ*(CRISPR) URA3::GPDp-*Os*Tir1-V5 HO::ADHp-3xHA-DHFR-3V5-OST(TM)-4aid-9myc::HYGR | This study |
| yPC12806 | Mata *ura3-52 his3D200 leu2D1 trp1D81* *cdc48-6* URA3::GPDp-*Os*Tir1-V5 HO::ADHp-3xHA-DHFR-3V5-OST(TM)-4aid-9myc::HYGR | This study |
| yPC12807 | Mata *ura3-52 his3D200 leu2D1 trp1D81* *cdc48-6 dfm1Δ*(CRISPR) URA3::GPDp-*Os*Tir1-V5 HO::ADHp-3xHA-DHFR-3V5-OST(TM)-4aid-9myc::HYGR | This study |
| yPC12812 | Mata *ura3-52 his3D200 leu2D1 trp1D81* cdc48^ΔHbYX^::HIS3 URA3::GPDp-*At*Tir1-9myc HO::ADHp-3xHA-DHFR-OST(TM)-4aid-9myc::HYGR | This study |
| yPC12813 | Mata *ura3-52 his3D200 leu2D1 trp1D81* cdc48^ΔHbYX^::HIS3 *dfm1Δ*(CRISPR) URA3::GPDp-*At*Tir1-9myc HO::ADHp-3xHA-DHFR-OST(TM)-4aid-9myc::HYGR | This study |
