# Supplementary material for: The derlin Dfm1 couples retrotranslocation of a folded protein domain to its proteasomal degradation
Source: J Cell Biol. 2024 Mar 5;223(5):e202308074. doi: 10.1083/jcb.202308074 (PMC11066878; doi:10.1083/jcb.202308074)
Supplement: Table S2 — lists plasmids used in this study. [file JCB_202308074_TableS2.docx]

**Table S2. List of plasmids used in this study**

| **Plasmid** | **Insert** | **Reference** |
| --- | --- | --- |
| PB565 | pRS425-Gal1p | This study |
| bPC204 | pDN201-GPDp-CPY*-HA | Ng et al., 2000 |
| bPC521 | pRS425-Gal1p_DFM1-FLAG | This study |
| bPC522 | pRS425-Gal1p_DFM1(WR-AA)-FLAG | This study |
| bPC1614 | pMK27-GPDp-*At*Tir1-9xMyc | Morawska et al., ‎2013 |
| bPC1680 | pFA6a-CPYp-3xHA-DHFR-OST1(TM)-4xAID-9xMyc-HphMX6 | This study |
| bPC1682 | pML107-dfm1Δ-gRNA1 | This study |
| bPC1696 | pML107-hrd1Δ-gRNA2 | This study |
| bPC1790 | pRS425-Gal1p_DFM1(GxxxG-AA)-FLAG | This study |
| bPC1798 | pFA6a-ADHp-3xHA-yeGFP-DHFR-OST1(TM)-4xAID-9xMyc-HphMX6 | This study |
| bPC1827 | pFA6a-ADHp-3xHA-DHFR-OST1(TM)-4xAID-9xMyc-HphMX6 | This study |
| bPC2016 | pFA6a-ADHp-3xHA-DHFR(29-31Pro)-OST1(TM)-4xAID-9xMyc-HphMX6 | This study |
| bPC2018 | pMK27-GPDp-*At*Tir1 | This study |
| bPC2172 | pRS415-ADHp-DFM1-FLAG | This study |
| bPC2179 | pRS415-ADHp-DFM1(GxxxG-AA)-FLAG | This study |
| bPC2282 | pRS415-ADHp-DFM1(WR-AA/GxxxG-AA)-FLAG | This study |
| bPC2284 | pML107-der1Δ-gRNA1 | This study |
| bPC2287 | pRS415-ADHp-DFM1(1-283)-FLAG | This study |
| bPC2328 | pRS415-ADHp-DFM1(WR-AA)-FLAG | This study |
| bPC2362 | pFA6a-ADHp-3xHA-DHFR-3xV5-OST1(TM)-4xAID-9xMyc-HphMX6 | This study |
| bPC2363 | pFA6a-ADHp-3xHA-DHFR-OST1(TM)-3xV5-4xAID-9xMyc-HphMX6 | This study |
| bPC2566 | pML107-pdr5Δ-gRNA1 | This study |
