# Supplementary material for: The derlin Dfm1 couples retrotranslocation of a folded protein domain to its proteasomal degradation
Source: J Cell Biol. 2024 Mar 5;223(5):e202308074. doi: 10.1083/jcb.202308074 (PMC11066878; doi:10.1083/jcb.202308074)
Supplement: Table S3 — lists oligonucleotides used in this study. [file JCB_202308074_TableS3.docx]

**Table S3. List of oligonucleotides used in this study**

| **Primer** | **Used for** | **Sequence** |
| --- | --- | --- |
| 4831 | Insert iRC variants into the HO locus (F) | GGCTTTTATTTCTATTACAACTATTAGCTCTAAATCCATATCCTCATAAGCAGCAATCAATTCTATCTATACGGCCGCCAGCTGAAGCTT |
| 4832 | Insert iRC variants into the HO locus (R) | CTAAAAATGGTTTTTTTCATCCAAAATATTAAATTTTACTTTTATTACATACAACTTTTTAAACTAATATATCGATGAATTCGAGCTCG |
| 3051 | Deletion of *HRD1* (F) | ACTGTCGACTTTCTACC |
| 3052 | Deletion of *HRD1* (R) | TCCGCTGATGTATAATATATATATATG |
| 131 | Deletion of *UBC7* (F) | GAGACAGGTAAAGTTATCCATTTTGAAATCATTAGGATTCGGTCGACGGATCCCCGGGTT |
| 132 | Deletion of *UBC7* (R) | TAAAAGGAAGACCAAATGATCATTAACCTGCTACCTGCTTTCGATGAATTCGAGCTCGTT |
| 245 | Deletion of *RAD23* (F) | CTAGGCAAGAAATAGCGACAG |
| 246 | Deletion of *RAD23* (R) | GTTCTTGAGCGATAATCCATTG |
| 247 | Deletion of *DSK2* (F) | CTAGTTCGGGACGTCTTAGTC |
| 248 | Deletion of *DSK2* (R) | GCCATTTAGCGTACGATATAC |
| 386 | Deletion *UBX2* (F) | GTATTACGATAGAAGTATGTAATAGCTTTCATAGTGTAATCGAAGCGGATCCCCGGGTTAATTAA |
| 59 | Deletion *UBX2* (R) | CGACAACCAATTGACCTTCAG |
| 251 | Deletion *UFD2* (F) | GTAGATTCTAACCATTGGCAAC |
| 3987 | Deletion *UFD2* (R) | CAACCAAAGGACTTGAGAGCCAAG |
| 3176 | Deletion *DER1* (F) | TATTCCTATAGTTTTGCTGAACATAG |
| 3177 | Deletion *DER1* (R) | TCCTTGGCCTATTTTATATGTAGTT |
| 3049 | Deletion *DFM1* (F) | AAGACAAGAGTATCAAACAAAGG |
| 3050 | Deletion *DFM1* (R) | GTTTCATTATCAATCGGTTGC |
| 3162 | Deletion *PDR5* (F) | TAAAAACTTATTATTACGCACCTATATG |
| 3163 | Deletion *PDR5* (R) | TTCTAATTATAAATAAATTGGCAACTAGG |
| 4808 | Deletion *PEP4* (F) | ATTTCATTTGCGGGTGTCG |
| 4809 | Deletion *PEP4* (R) | CTTTATTATCAATTGTTCCGCTATTGG |
| 364 | Generation cdc48^ΔHbYX^ (F) | CAGGTGCTGCATTTGGTTCTAATGCGGAGGAAGATGATGATTGAGGCGCGCCACTTCTAAA |
| 53 | Generation cdc48^ΔHbYX^ (R) | TGAATTTACGATTTAAAATAAAAATATACCTGGCATATAATCGATGAATTCGAGCTCGTT |
| 2995 | Cloning DFM1-FLAG in pRS425-GALp (F) | GAACTAGTGGATCCAATACCATGGCAGGCCCAAGGAATG |
| 2996 | Cloning DFM1-FLAG in pRS425-GALp (R) | GGGCCTGCCATGGTATTGGATCCACTAGTTC |
| 2997 | Cloning DFM1(1-283)-FLAG in pRS425-GALp (F) | GAGAAACGAGAACTGGTTCGGGTGAAAACTTGTAC |
| 2998 | Cloning DFM1(1-283)-FLAG in pRS425-GALp (R) | CAAGTTTTCACCCGAACCAGTTCTCGTTTCTCTTTGCG |
| 3187 | Mutagenesis for DFM1-(WR-AA) (F) | TTGAATCTAATCAATCCCTGGTATTTCATTTACGTATGGAATTTGACGTTCAAGAAGGTTCAGATAGCGGCACTTCTTACTTCTTGTGTA |
| 3190 | Mutagenesis for DFM1-(WR-AA) (R) | TCATAAATACTATATAGTTCCATTAGCGCAGGCATGGCACGAGACGAAAGCATTACACAAGAAGTAAGAAGTGCCGCTATCTGAACCTTC |
| 3202 | Mutagenesis for DFM1-(GXXXG-AA) (F) | AGGTACGCTGTAGTAAAAGCGATCAATGAGATTACGAA |
| 3203 | Mutagenesis for DFM1-(GXXXG-AA) (R) | GATCGCTTTTACTACAGCGTACCTTTATACATGCTT |
| 3165 | Mutagenesis for iRC(Pro) (F) | CCTGCCTGCCGATCTCCCACCTCCAAAACGCAACACCTTA |
| 3166 | Mutagenesis for iRC(Pro) (R) | TAAGGTGTTGCGTTTTGGAGGTGGGAGATCGGCAGGCAGG |
| 4885 | Cloning (Lum linker)-iRC (F) | CCCGGGCCCAGCGGC |
| 4862 | Cloning (Lum linker)-iRC (R) | CCCCGTACGGTTAGTTACGTTCATGTTCAAAGTTTTTAAGACAAAAAC |
| 4848 | Cloning (Cyt linker)-iRC (F) | CCCGGGCCCAGCGGCTCTGGCCAAG |
| 4886 | Cloning (Cyt linker)-iRC (R) | GGGcgtacgAGTGGAATCTAATCCC |
