# Supplementary material for: The derlin Dfm1 couples retrotranslocation of a folded protein domain to its proteasomal degradation
Source: J Cell Biol. 2024 Mar 5;223(5):e202308074. doi: 10.1083/jcb.202308074 (PMC11066878; doi:10.1083/jcb.202308074)
Supplement: Table S4 — lists antibodies used in this study. [file JCB_202308074_TableS4.docx]

**Table S4. List of antibodies used in this study**

| **Antibody** | **Dilution** | **Source** |
| --- | --- | --- |
| rat monoclonal anti-HA (3F10) | 1:2000 | Roche - Cat# 11867423001 |
| mouse monoclonal anti-Pgk1 | 1:10000 | Invitrogen - Cat# 459250 |
| rabbit polyclonal anti-Kar2 | 1:1000 | Santa Cruz - Cat# sc-33630 |
| mouse monoclonal anti-Dpm1 (5C5A7) | 1:10000 | Life Technologies -  Cat# A6429 |
| rabbit polyclonal anti-Erg1 | 1:3000 | raised |
| rabbit polyclonal anti-Dfm1 | 1:1000 | raised |
| rabbit polyclonal anti-Cdc48 | 1:1000 | gift from Y. Ye |
| rabbit polyclonal anti-Asi2 | 1:5000 | Natarajan et al., Mol Cell 2020 |
| mouse monoclonal anti-Pdi1 | 1:10000 | Invitrogen -  Cat# MA1-10032 |
| rabbit monoclonal anti-V5 (D3H8Q) | 1: 5000 | Cell Signaling - Cat# 13202S |
| rabbit polyclonal anti-Usa1 | 1:3000 | Carvalho et al., Cell 2006 |
| mouse monoclonal anti-myc (9E10) | 1:1000 | Roche - Cat# 11667203001 |
| mouse monoclonal anti-FLAG-HRP clone M2 | 1:2000 | Sigma-Aldrich - Cat# A8592 |
| rabbit monoclonal anti-Ufd2 | 1:500 | Richly et al., Cell 2005 |
| rabbit polyclonal anti-Shp1 | 1:10000 | Rumpf et al., Mol Cell 2006 |
| rabbit polyclonal anti-Ufd1 | 1:2000 | Rumpf et al., Mol Cell 2006 |
| rabbit polyclonal anti-Otu1 | 1:1000 | Rumpf et al., Mol Cell 2006 |
