# Supplementary figures and images for: The derlin Dfm1 couples retrotranslocation of a folded protein domain to its proteasomal degradation
Source: J Cell Biol. 2024 Mar 5;223(5):e202308074. doi: 10.1083/jcb.202308074 (PMC11066878; doi:10.1083/jcb.202308074)

Fig. 1B

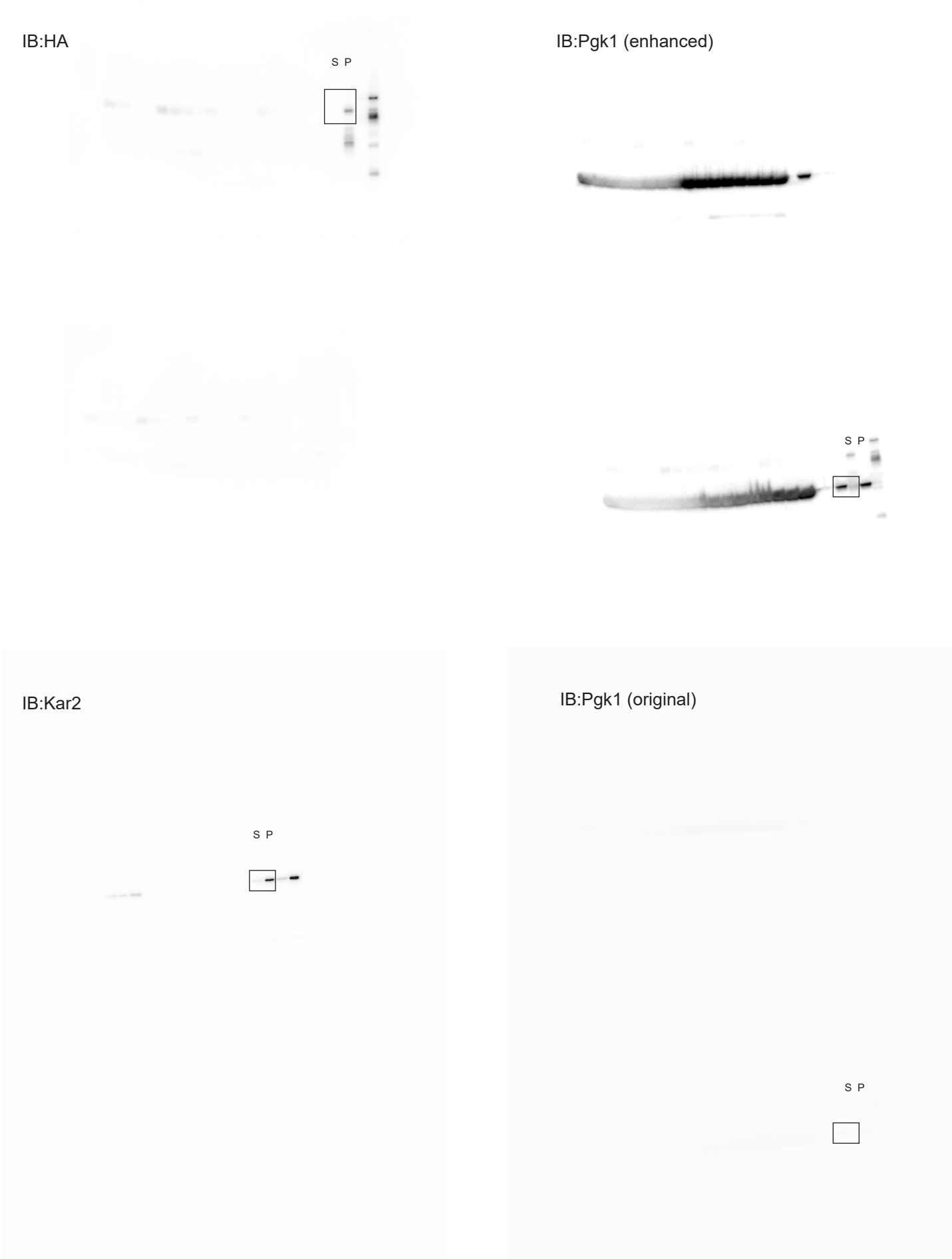

Fig. 1D

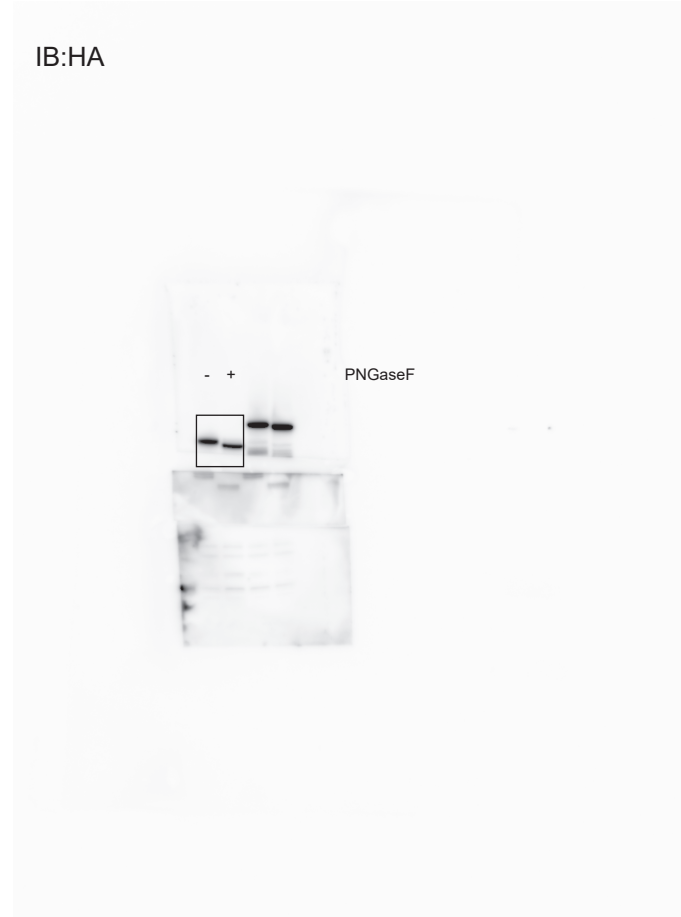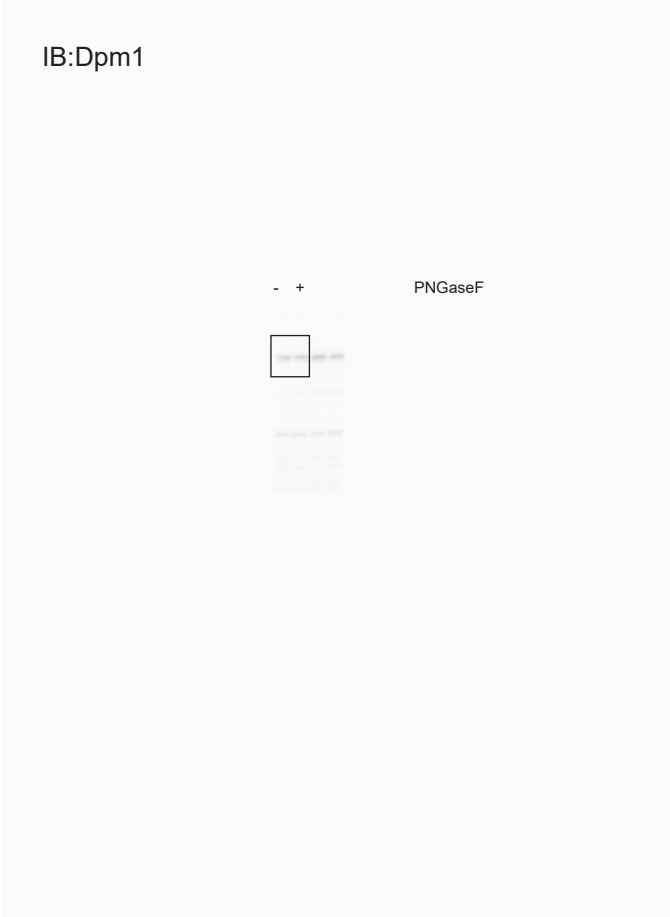

Fig. 1F

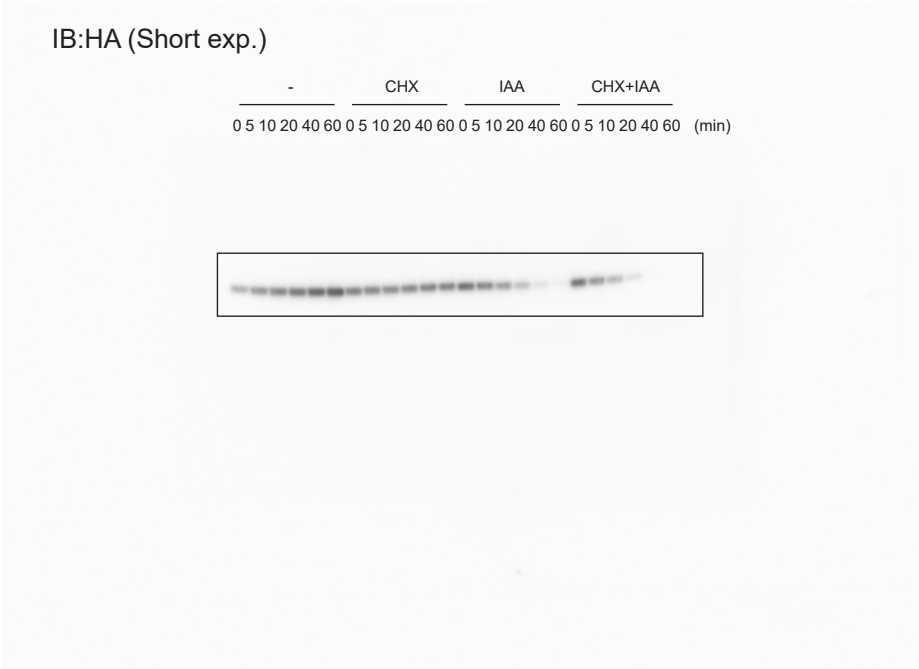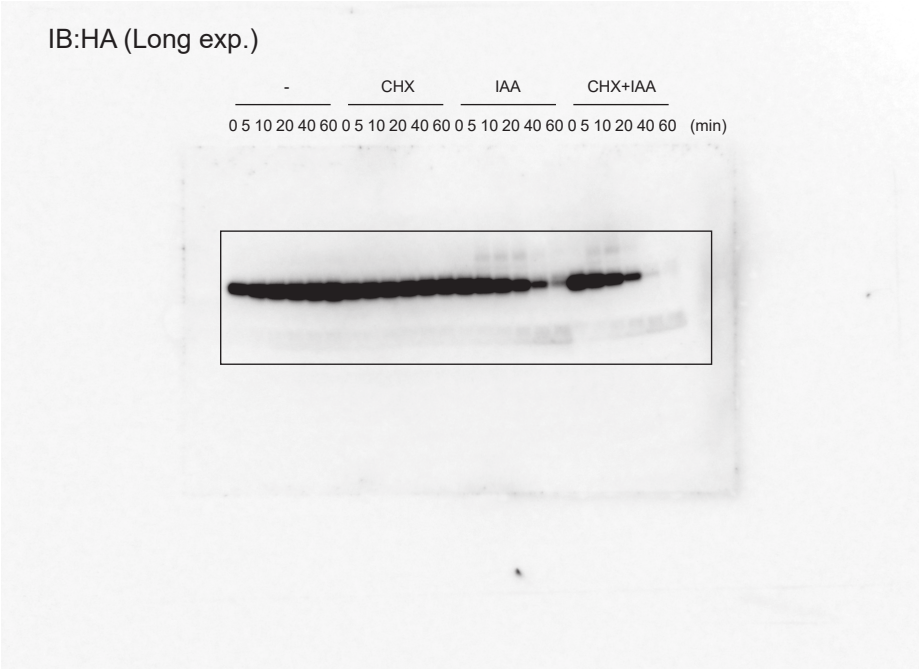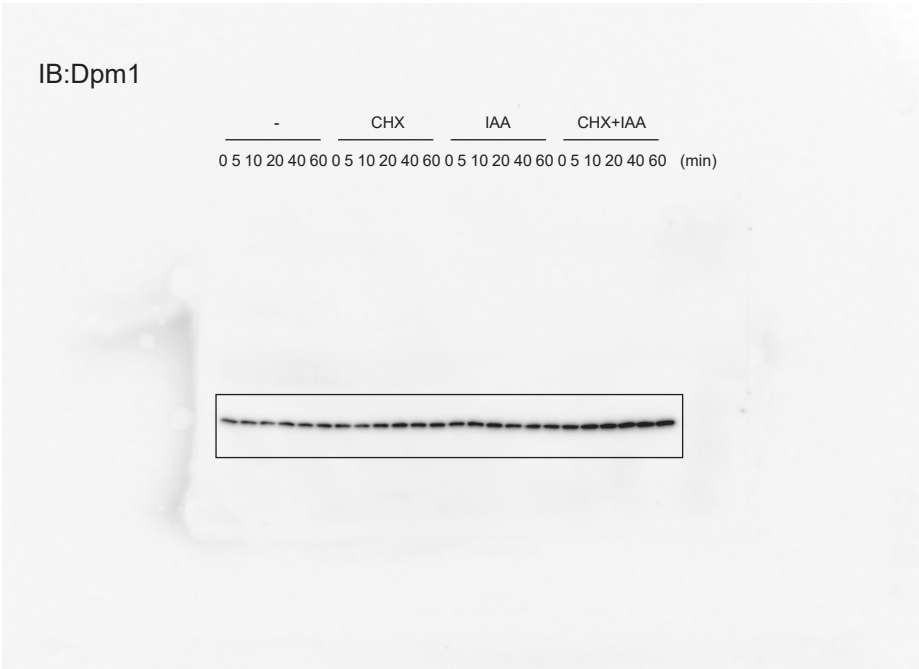

Fig. 1G

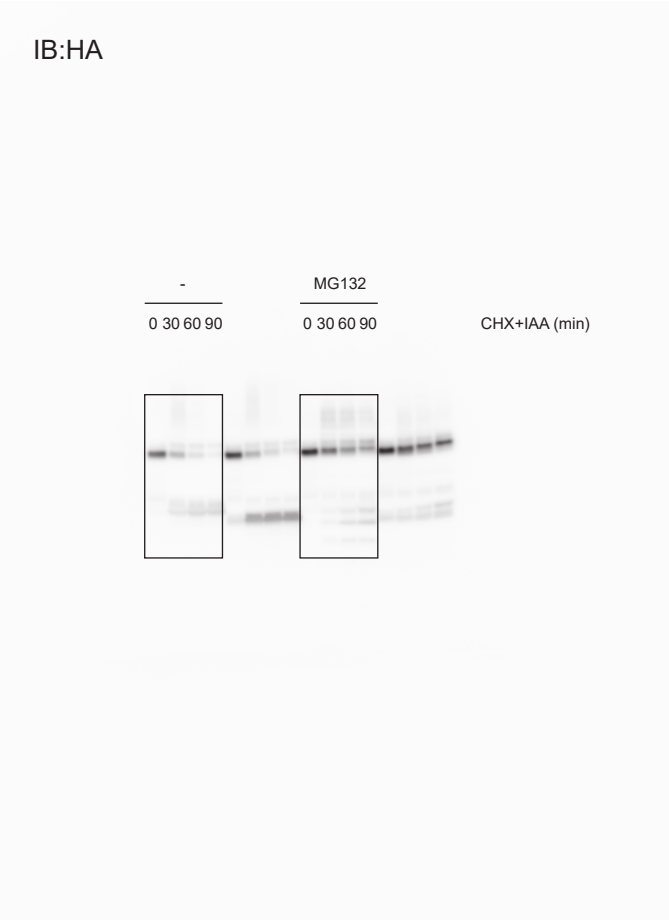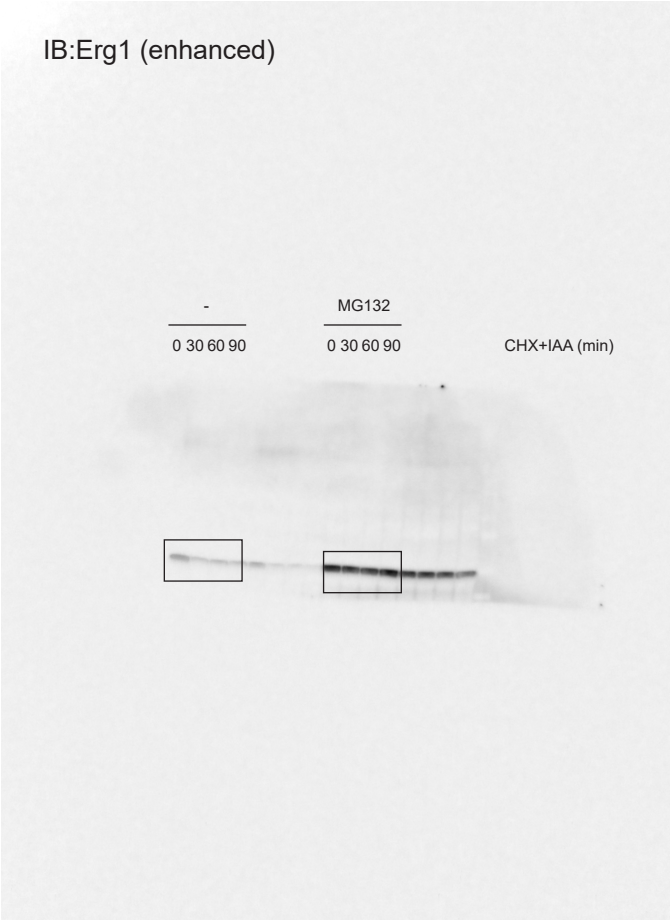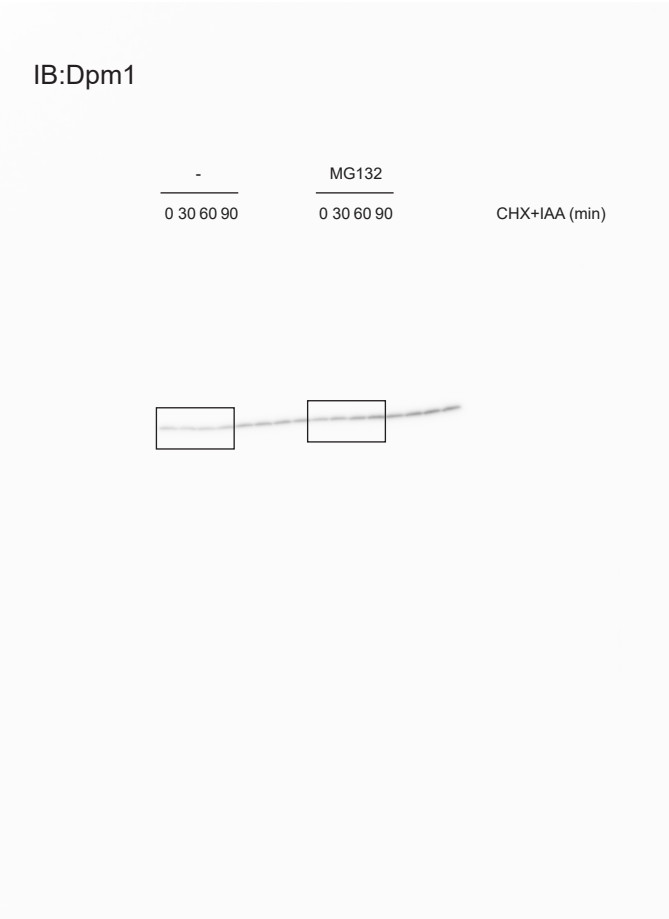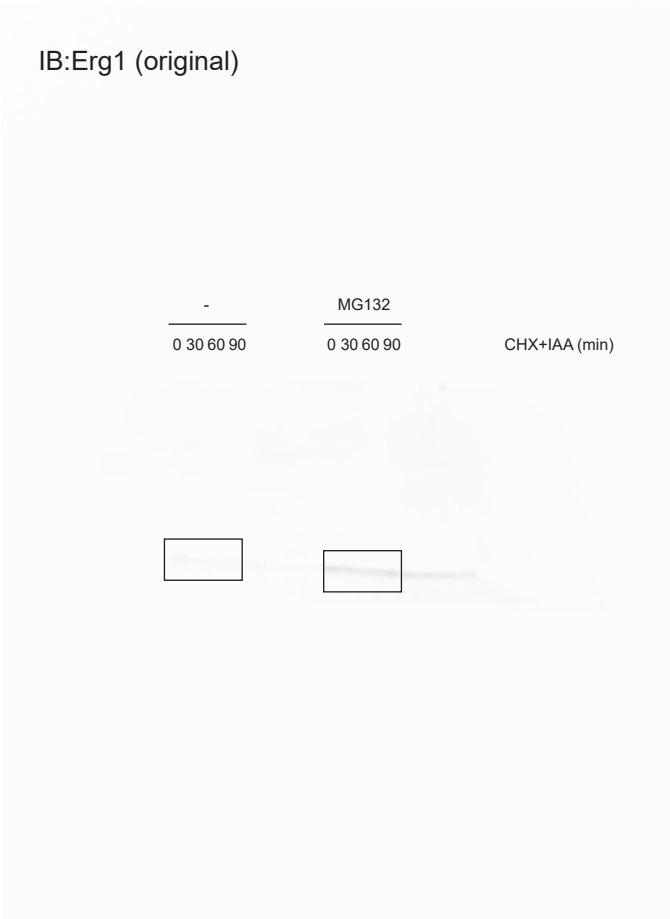

Supplement: SourceData F1 — is the source file for Fig. 1. [file JCB_202308074_SourceDataF1.pdf]

Fig. 3A

IB:HA (25°C)

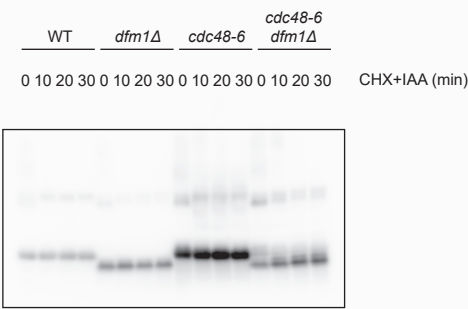

IB:Dpm1 (25°C)

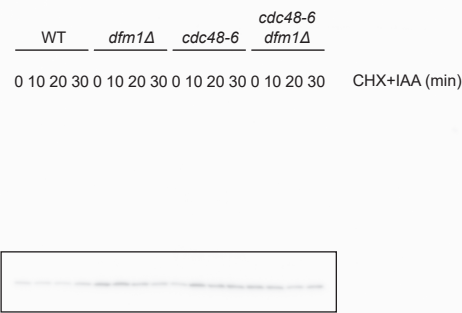

IB:HA (37°C)

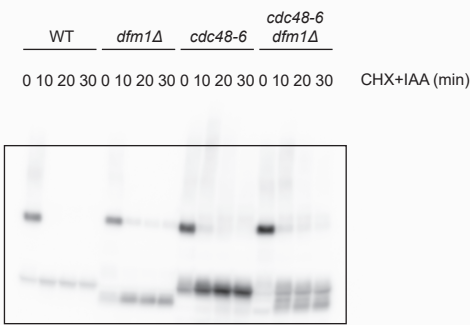

IB:Dpm1 (37°C)

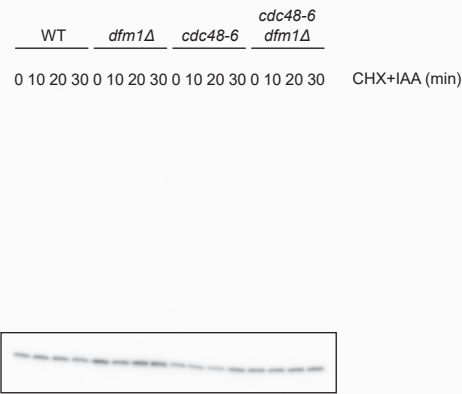

Fig. 3B

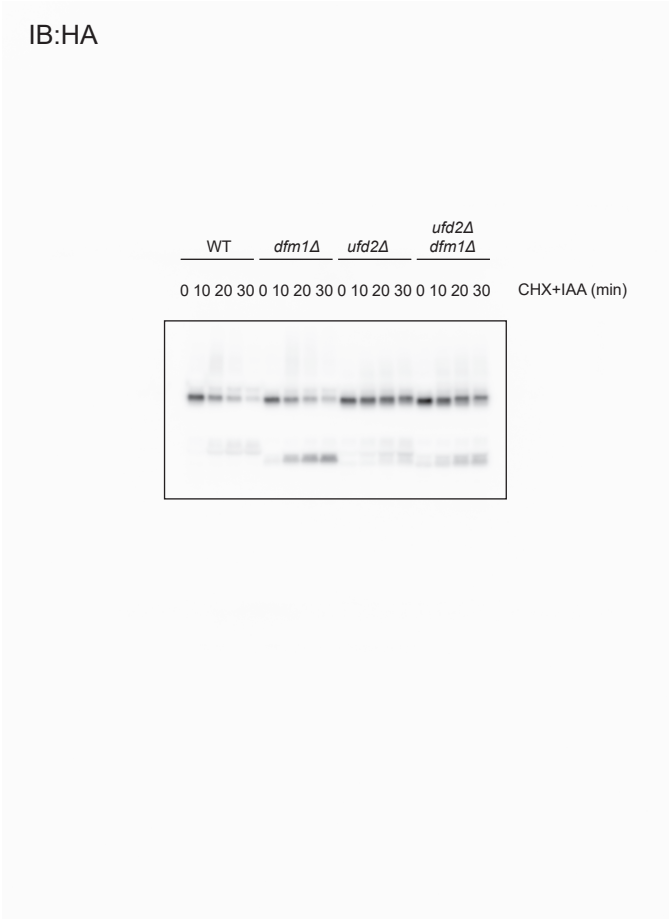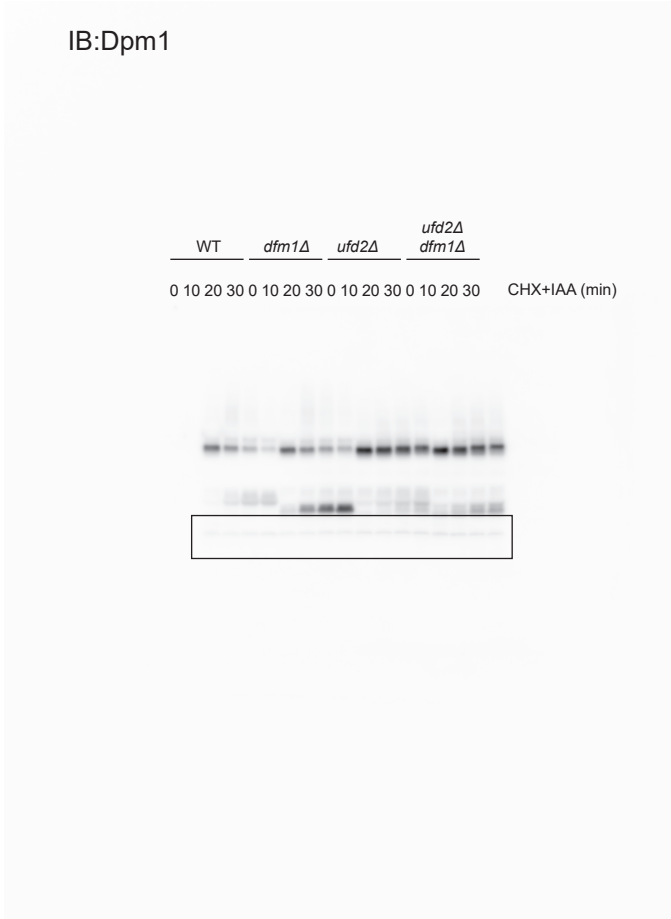

Fig. 3C

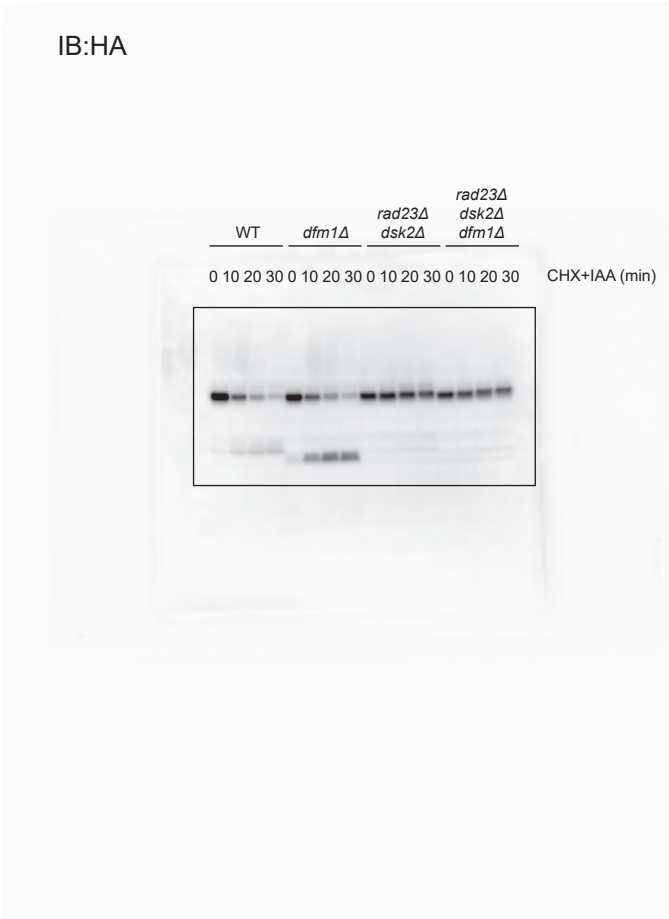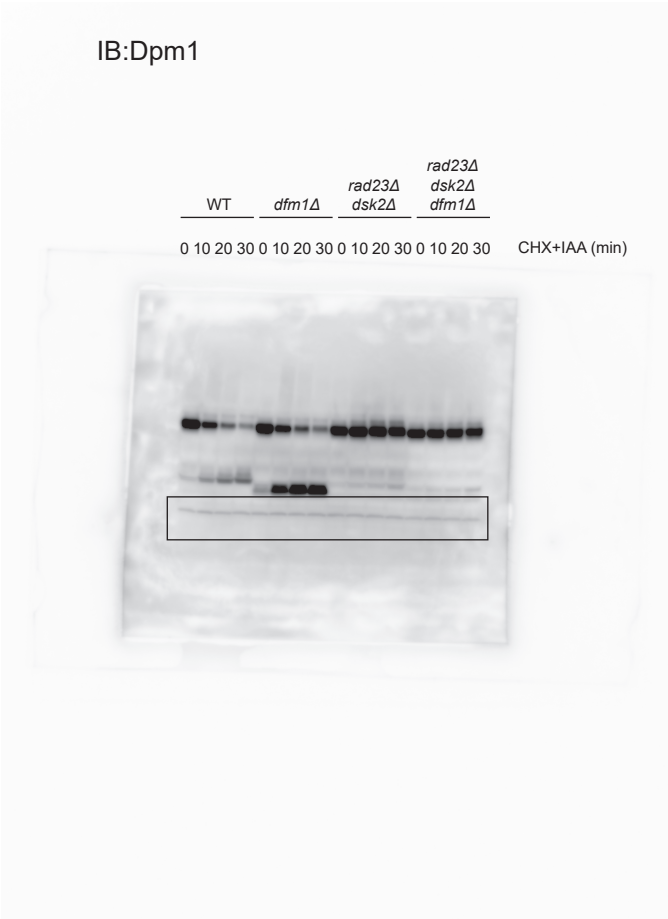

Fig. 3D

IB:HA

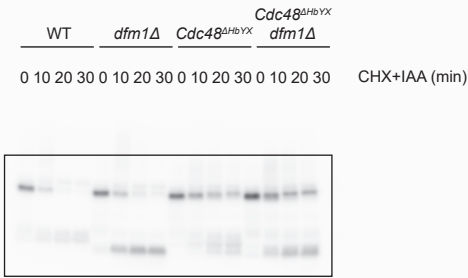

IB:Cdc48

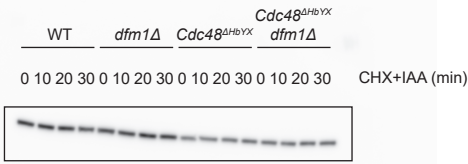

IB:Erg1

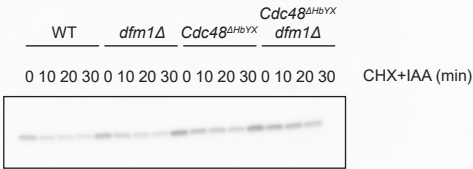

IB:Dpm1

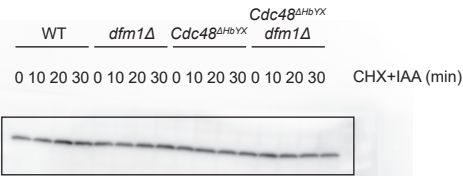

Supplement: SourceData F3 — is the source file for Fig. 3. [file JCB_202308074_SourceDataF3.pdf]

Fig. 4A

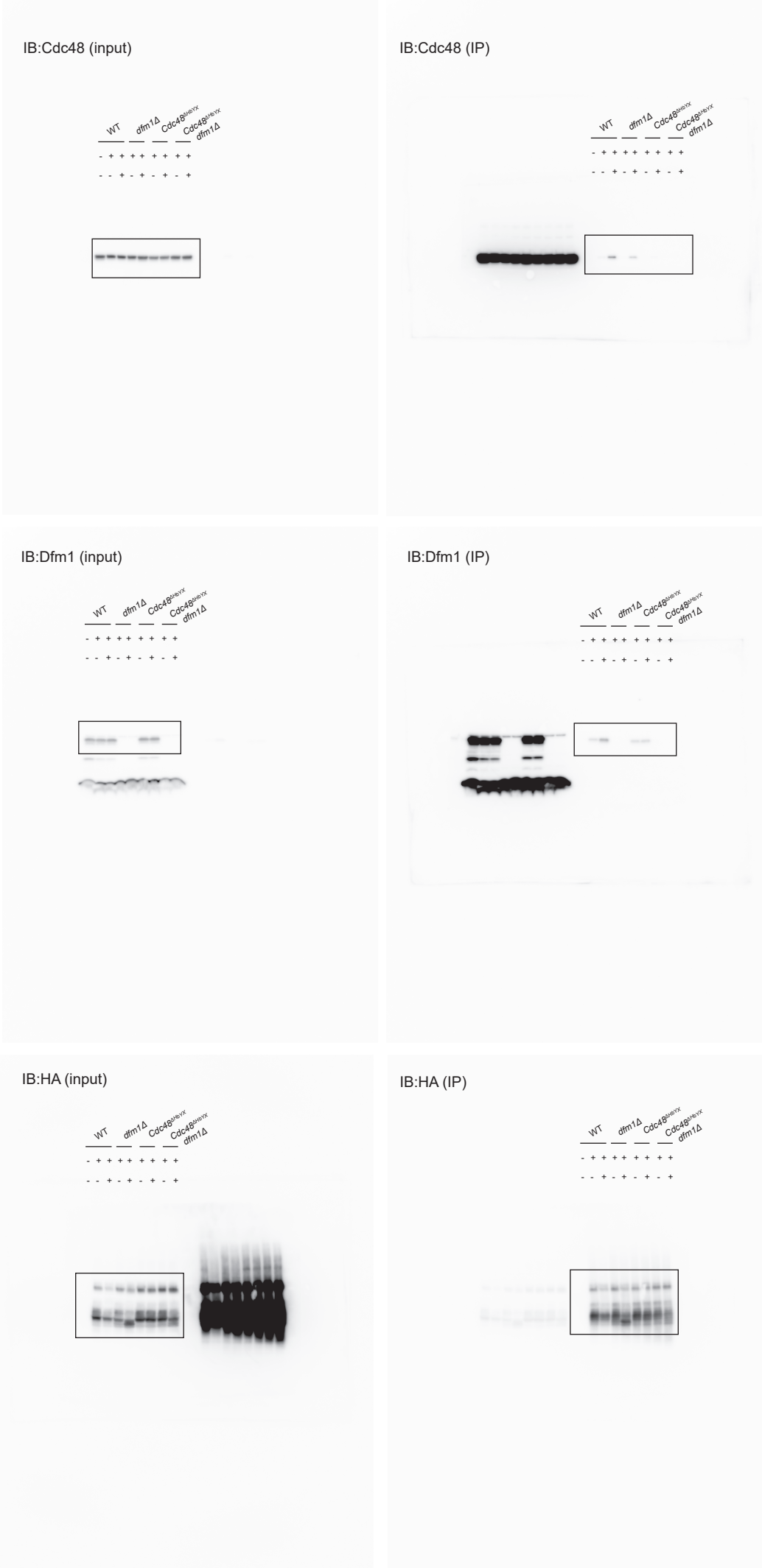

Fig. 4B

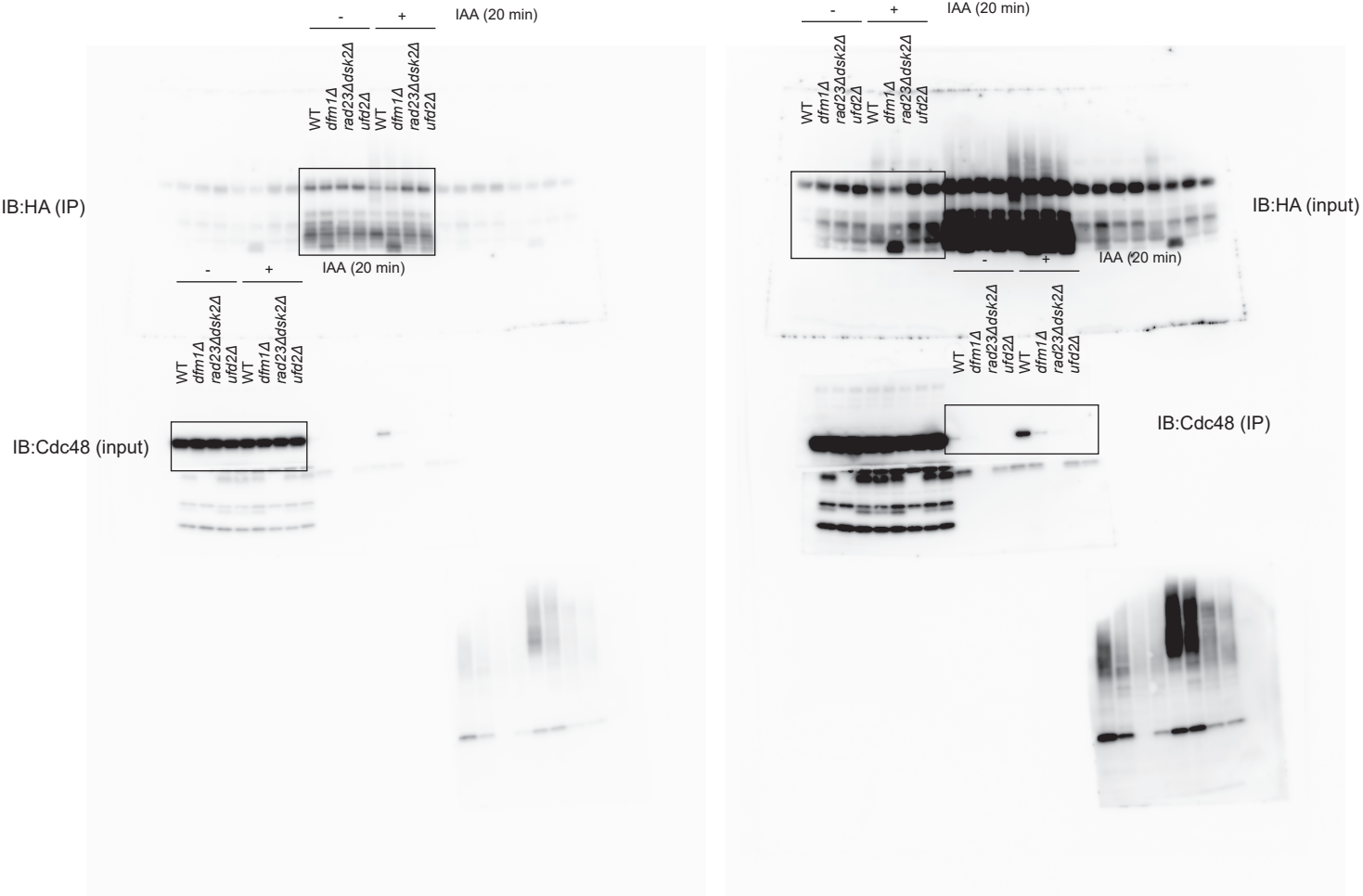

Supplement: SourceData F4 — is the source file for Fig. 4. [file JCB_202308074_SourceDataF4.pdf]

Fig. S2

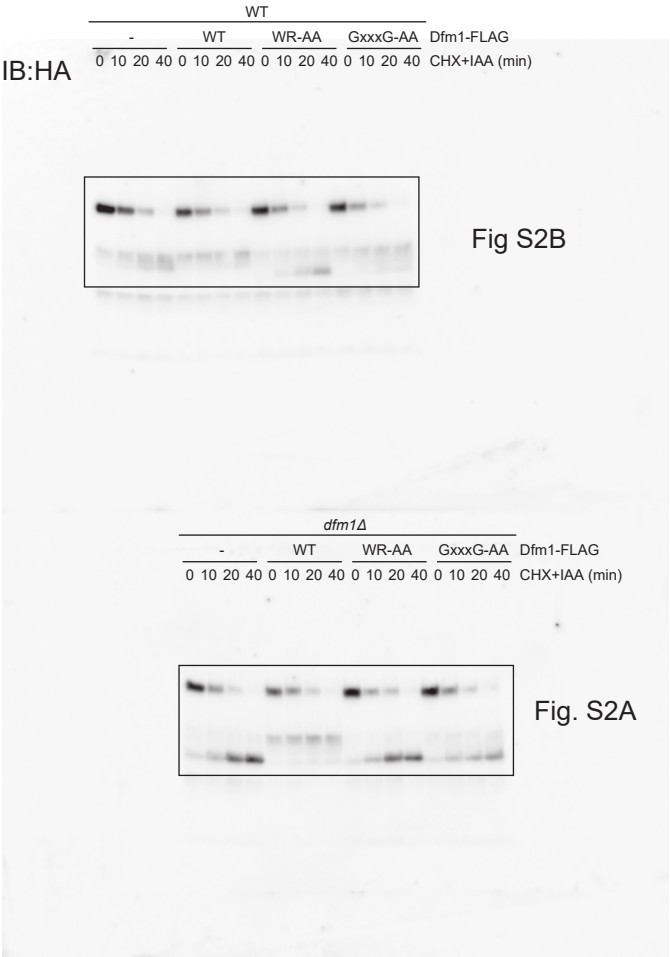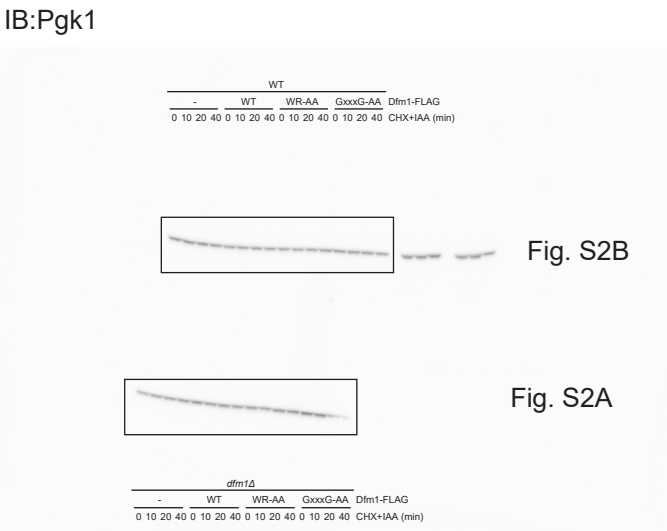

IB:Flag

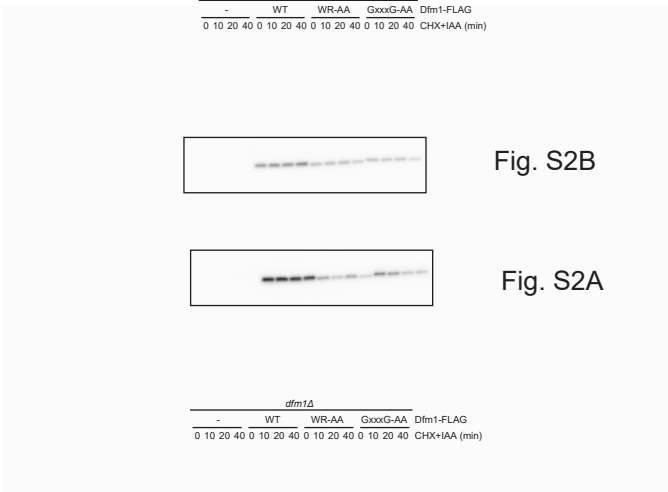

Supplement: SourceData FS2 — is the source file for Fig. S2. [file JCB_202308074_SourceDataFS2.pdf]

Fig. S3A

IB:HA

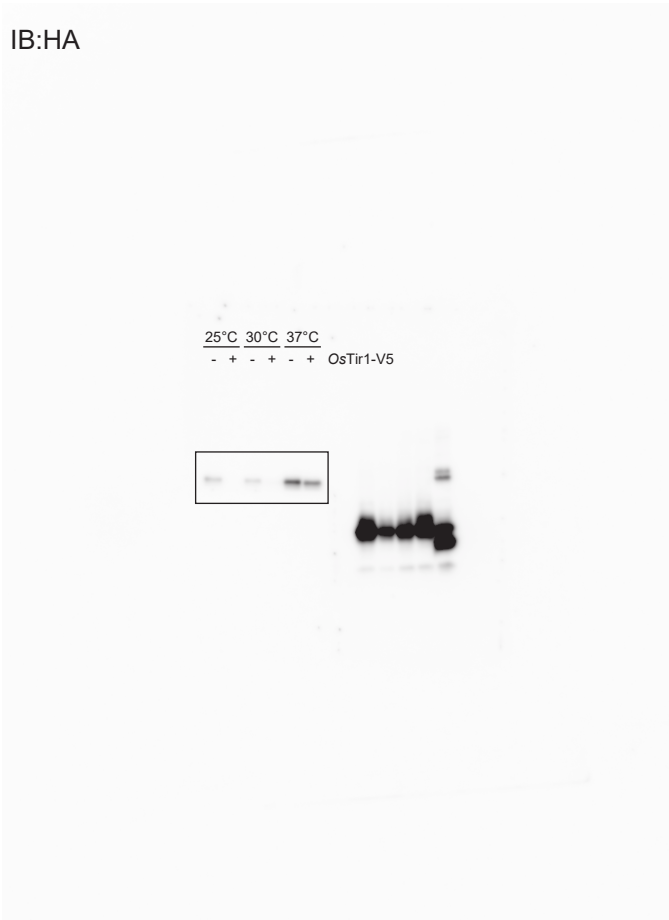

IB:V5

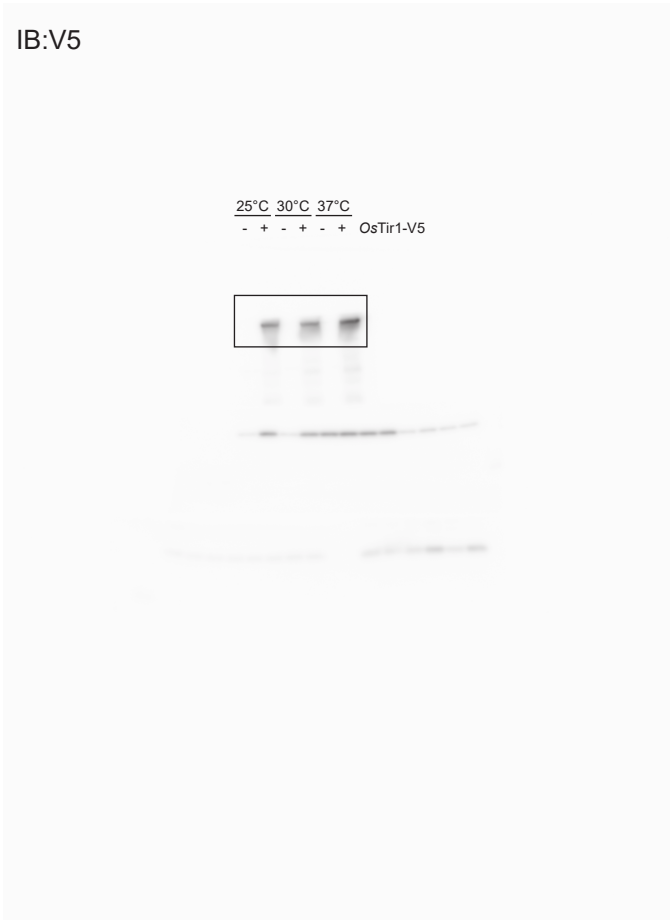

IB:Dpm1 (original)

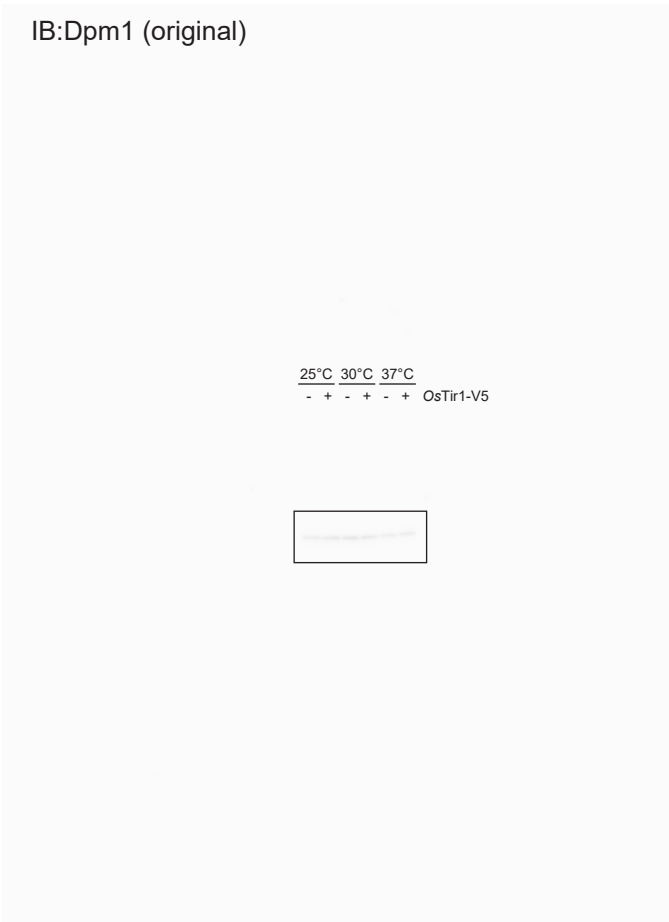

IB:Dpm1 (enhanced)

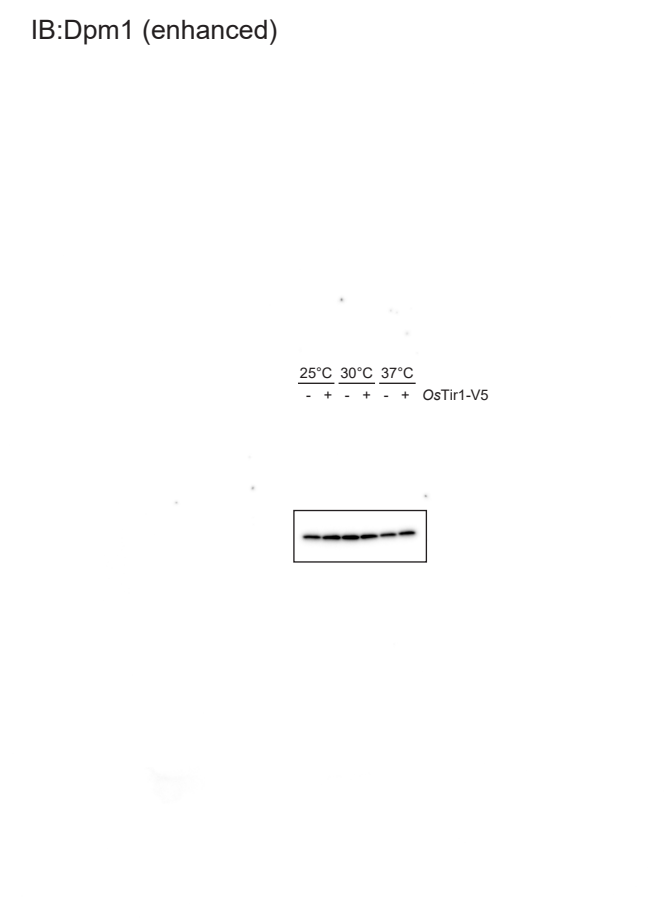

Fig. S3B

IB:HA

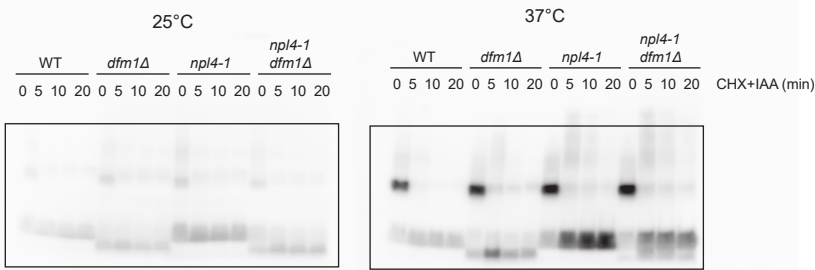

IB:Dpm1 (37°C)

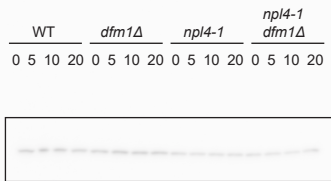

IB:Dpm1 (25°C)

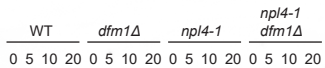

Fig. S3C

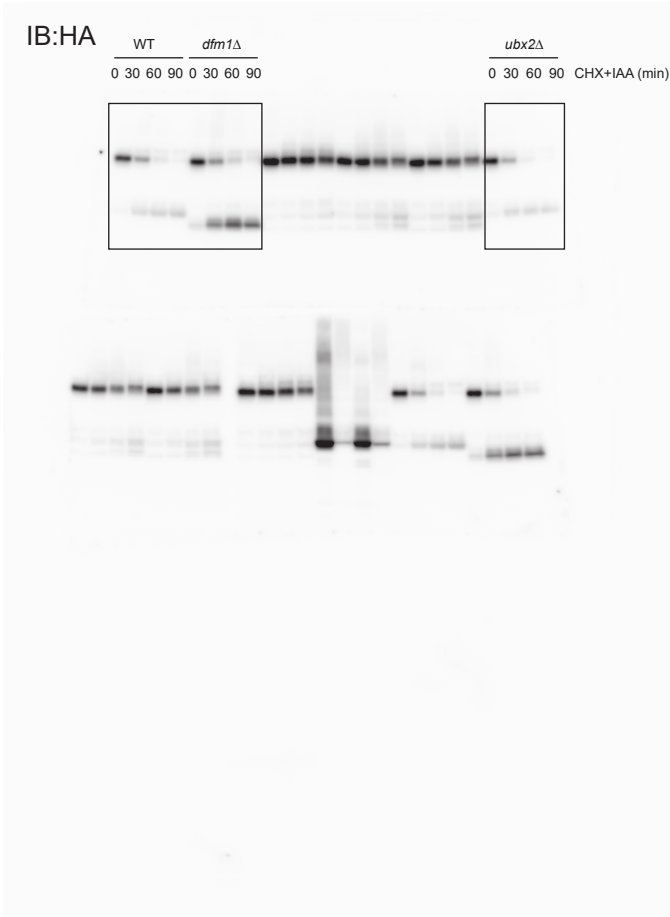

Supplement: SourceData FS3 — is the source file for Fig. S3. [file JCB_202308074_SourceDataFS3.pdf]
