# Supplementary material for: The derlin Dfm1 couples retrotranslocation of a folded protein domain to its proteasomal degradation
Source: J Cell Biol. 2024 Mar 5;223(5):e202308074. doi: 10.1083/jcb.202308074 (PMC11066878; doi:10.1083/jcb.202308074)
Supplement: SourceData F2 — is the source file for Fig. 2. [file JCB_202308074_SourceDataF2.pdf]

Fig. 2A

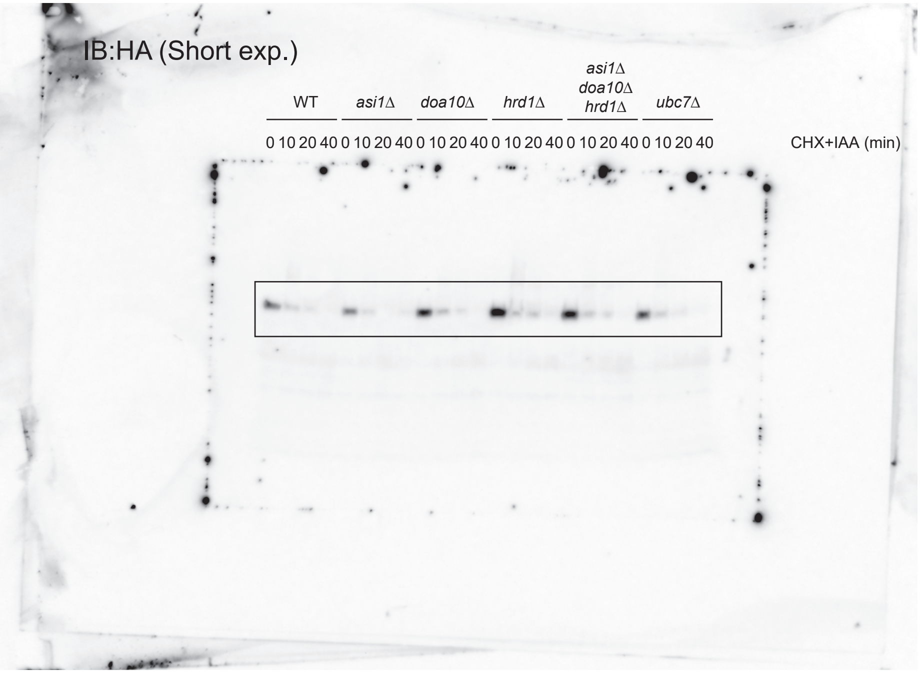
IB:HA (Short exp.)
WT
asi1Δ
doa10Δ
hrd1Δ
asi1Δ doa10Δ hrd1Δ
ubc7Δ
0 10 20 40 0 10 20 40 0 10 20 40 0 10 20 40 0 10 20 40 0 10 20 40
CHX+IAA (min)

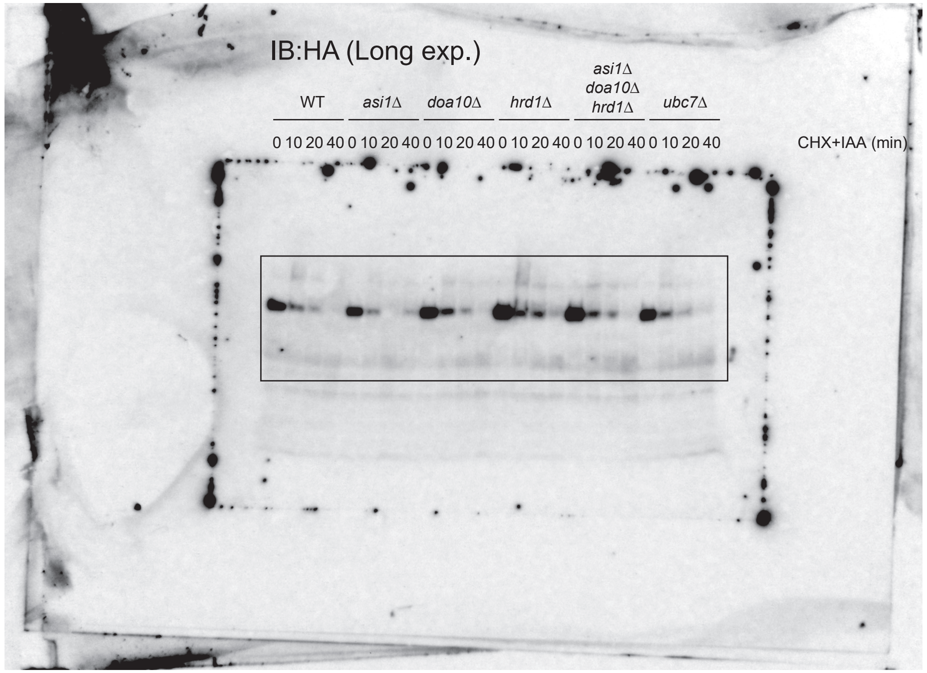
IB:HA (Long exp.)
WT
asi1Δ
doa10Δ
hrd1Δ
asi1Δ doa10Δ hrd1Δ
ubc7Δ
0 10 20 40 0 10 20 40 0 10 20 40 0 10 20 40 0 10 20 40 0 10 20 40
CHX+IAA (min)

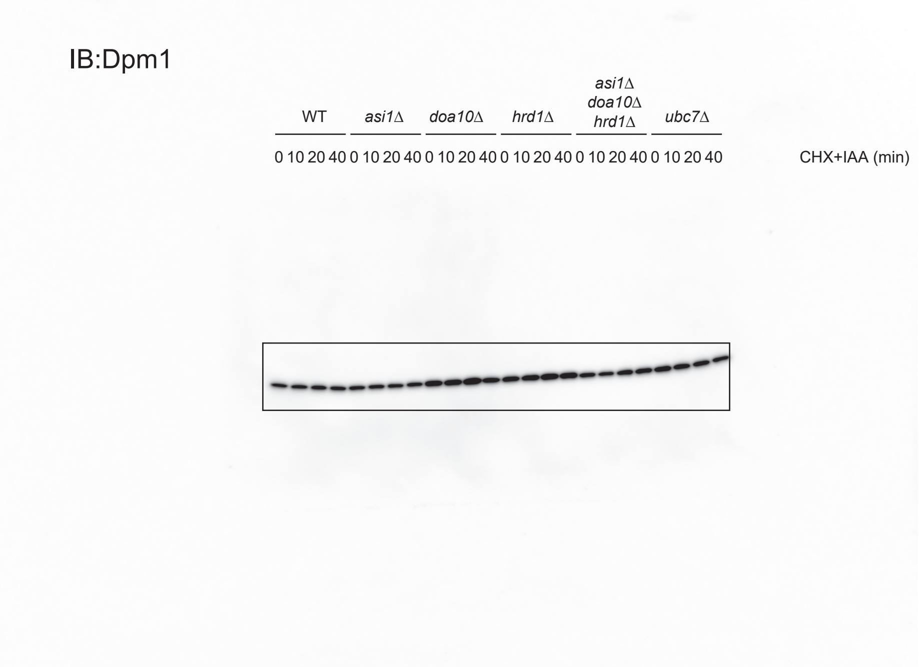
IB:Dpm1
WT
asi1Δ
doa10Δ
hrd1Δ
asi1Δ doa10Δ hrd1Δ
ubc7Δ
0 10 20 40 0 10 20 40 0 10 20 40 0 10 20 40 0 10 20 40 0 10 20 40
CHX+IAA (min)

Fig. 2B

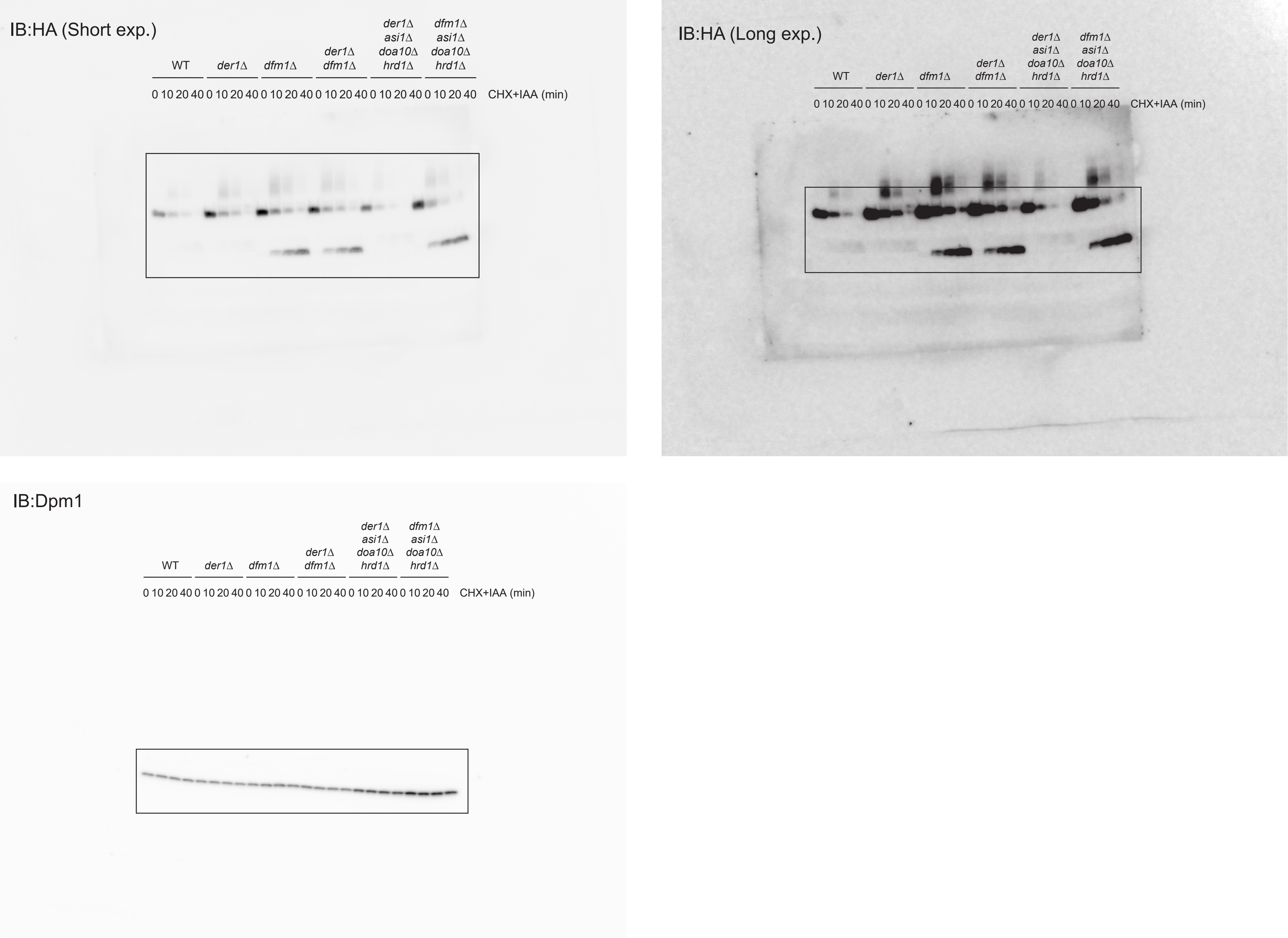
IB:HA (Short exp.)
WT
der1Δ
dfm1Δ
der1Δ dfm1Δ
der1Δ asi1Δ doa10Δ hrd1Δ
dfm1Δ asi1Δ doa10Δ hrd1Δ
0 10 20 40 0 10 20 40 0 10 20 40 0 10 20 40 0 10 20 40 0 10 20 40
CHX+IAA (min)
IB:HA (Long exp.)
WT
der1Δ
dfm1Δ
der1Δ dfm1Δ
der1Δ asi1Δ doa10Δ hrd1Δ
dfm1Δ asi1Δ doa10Δ hrd1Δ
0 10 20 40 0 10 20 40 0 10 20 40 0 10 20 40 0 10 20 40 0 10 20 40
CHX+IAA (min)
IB:Dpm1
WT
der1Δ
dfm1Δ
der1Δ dfm1Δ
der1Δ asi1Δ doa10Δ hrd1Δ
dfm1Δ asi1Δ doa10Δ hrd1Δ
0 10 20 40 0 10 20 40 0 10 20 40 0 10 20 40 0 10 20 40 0 10 20 40
CHX+IAA (min)

Fig. 2D

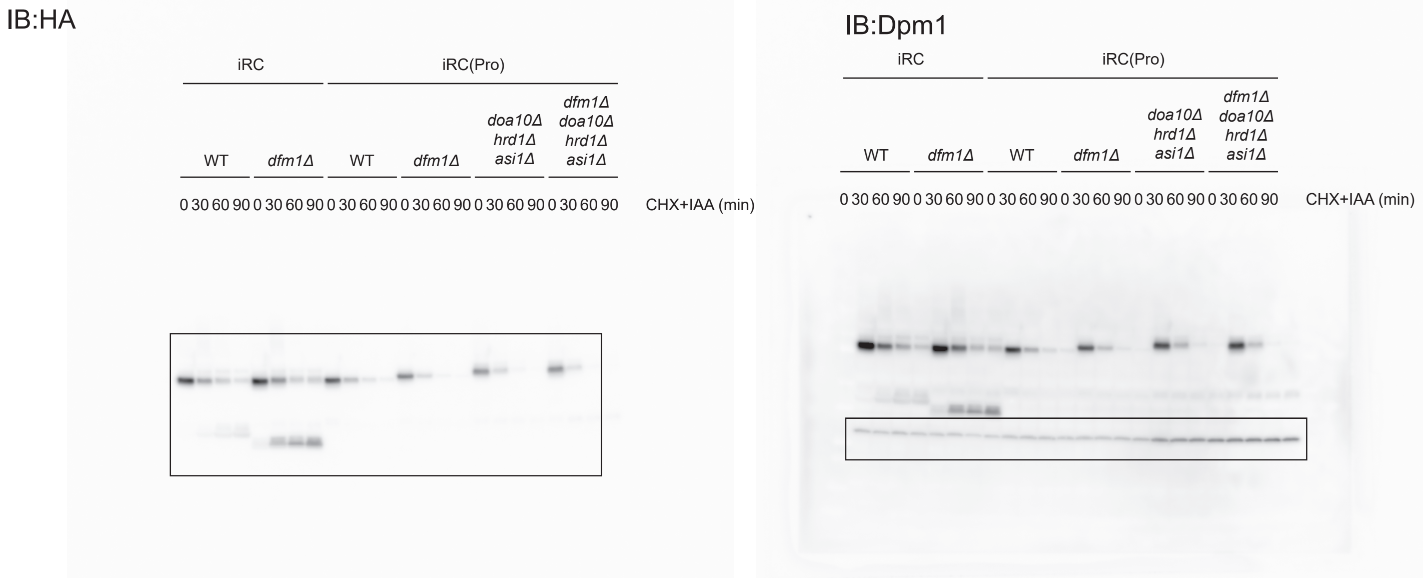

IB:HA
iRC
iRC(Pro)
WT
dfm1Δ
WT
dfm1Δ
doa10Δ hrd1Δ asi1Δ
dfm1Δ doa10Δ hrd1Δ asi1Δ
0 30 60 90 0 30 60 90 0 30 60 90 0 30 60 90 0 30 60 90 0 30 60 90
CHX+IAA (min)
IB:Dpm1
iRC
iRC(Pro)
WT
dfm1Δ
WT
dfm1Δ
doa10Δ hrd1Δ asi1Δ
dfm1Δ doa10Δ hrd1Δ asi1Δ
0 30 60 90 0 30 60 90 0 30 60 90 0 30 60 90 0 30 60 90 0 30 60 90
CHX+IAA (min)

Fig. 2E

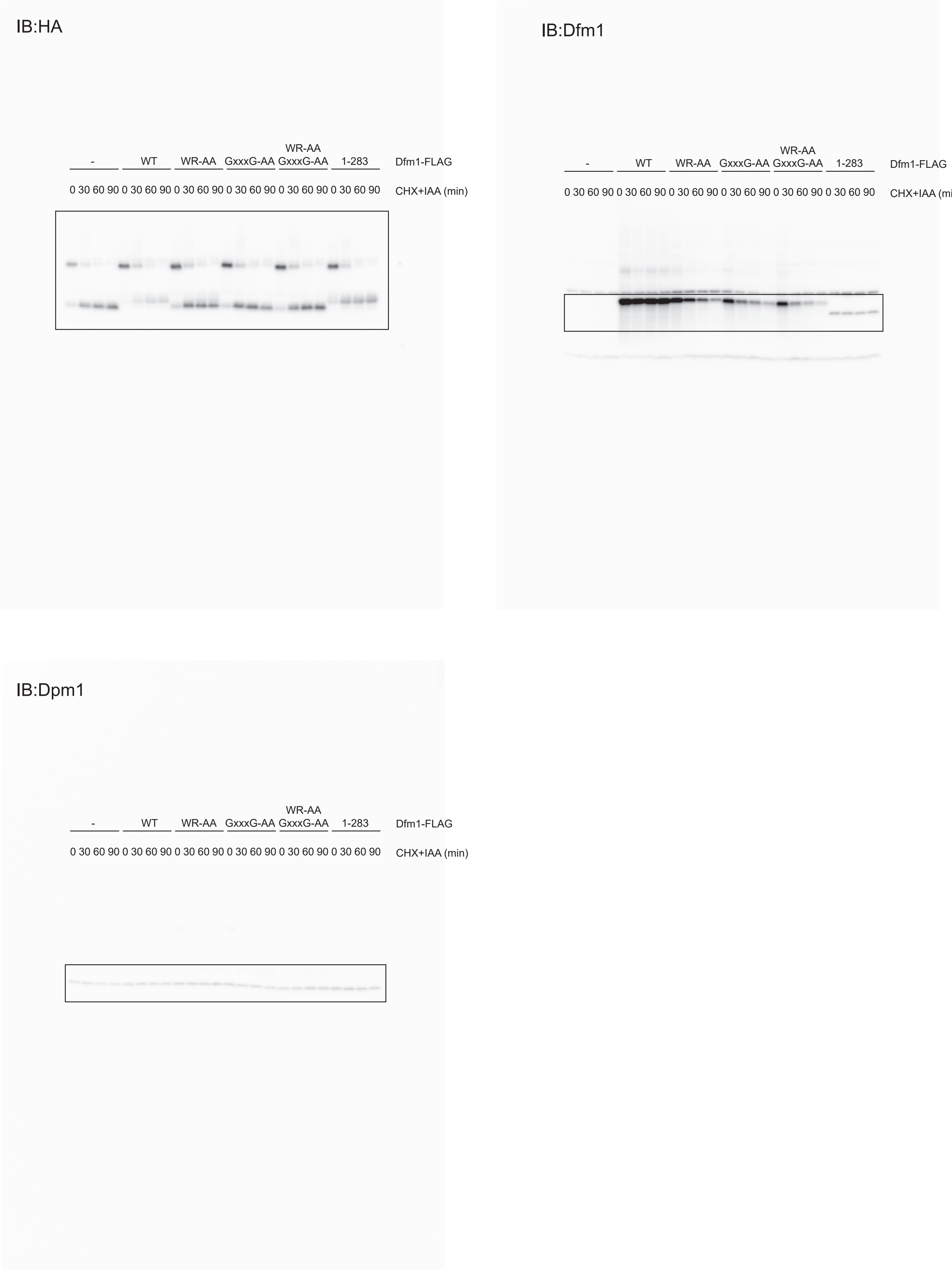

IB:HA
- WT WR-AA GxxxG-AA WR-AA GxxxG-AA 1-283 Dfm1-FLAG
0 30 60 90 0 30 60 90 0 30 60 90 0 30 60 90 0 30 60 90 0 30 60 90 CHX+IAA (min)
IB:Dfm1
- WT WR-AA GxxxG-AA WR-AA GxxxG-AA 1-283 Dfm1-FLAG
0 30 60 90 0 30 60 90 0 30 60 90 0 30 60 90 0 30 60 90 0 30 60 90 CHX+IAA (min)
IB:Dpm1
- WT WR-AA GxxxG-AA WR-AA GxxxG-AA 1-283 Dfm1-FLAG
0 30 60 90 0 30 60 90 0 30 60 90 0 30 60 90 0 30 60 90 0 30 60 90 CHX+IAA (min)
