# Supplementary material for: The derlin Dfm1 couples retrotranslocation of a folded protein domain to its proteasomal degradation
Source: J Cell Biol. 2024 Mar 5;223(5):e202308074. doi: 10.1083/jcb.202308074 (PMC11066878; doi:10.1083/jcb.202308074)
Supplement: SourceData F5 — is the source file for Fig. 5. [file JCB_202308074_SourceDataF5.pdf]

Fig. 5A

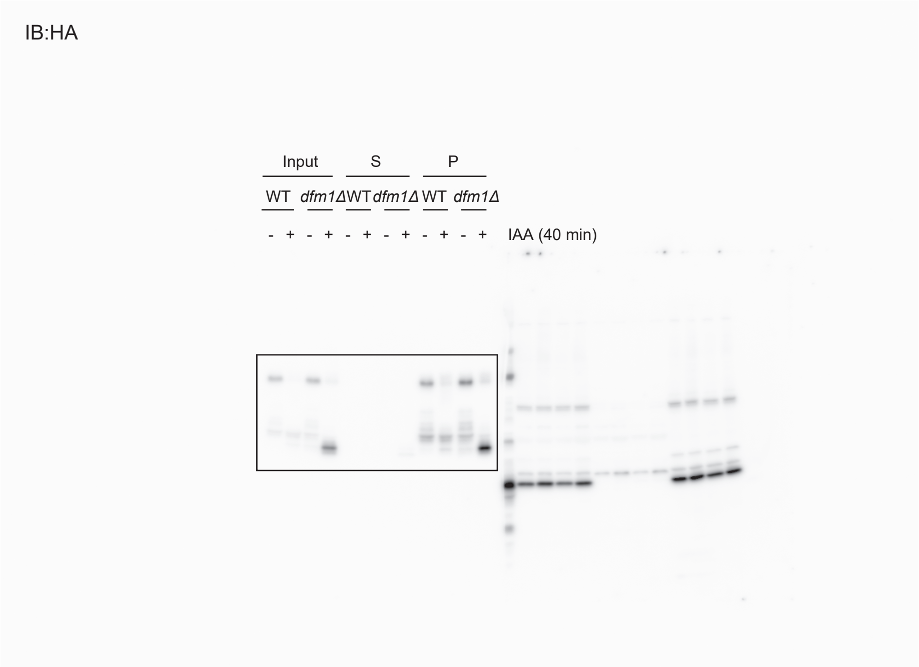
IB:HA
Input
S
P
WT
dfm1Δ
WT
dfm1Δ
WT
dfm1Δ
- + - + - + - + - + - +
IAA (40 min)

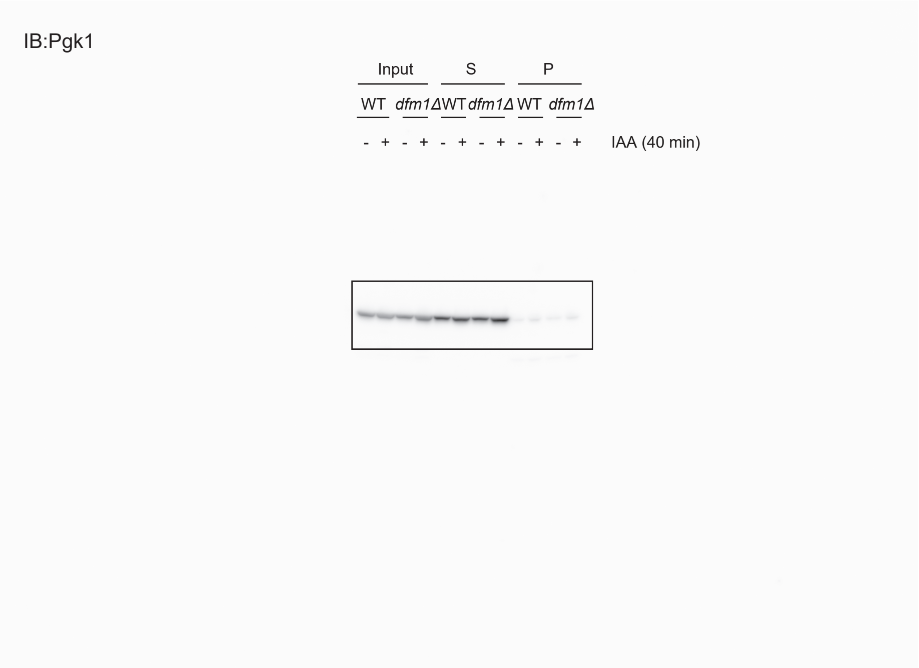
IB:Pgk1
Input
S
P
WT
dfm1Δ
WT
dfm1Δ
WT
dfm1Δ
- + - + - + - + - + - +
IAA (40 min)

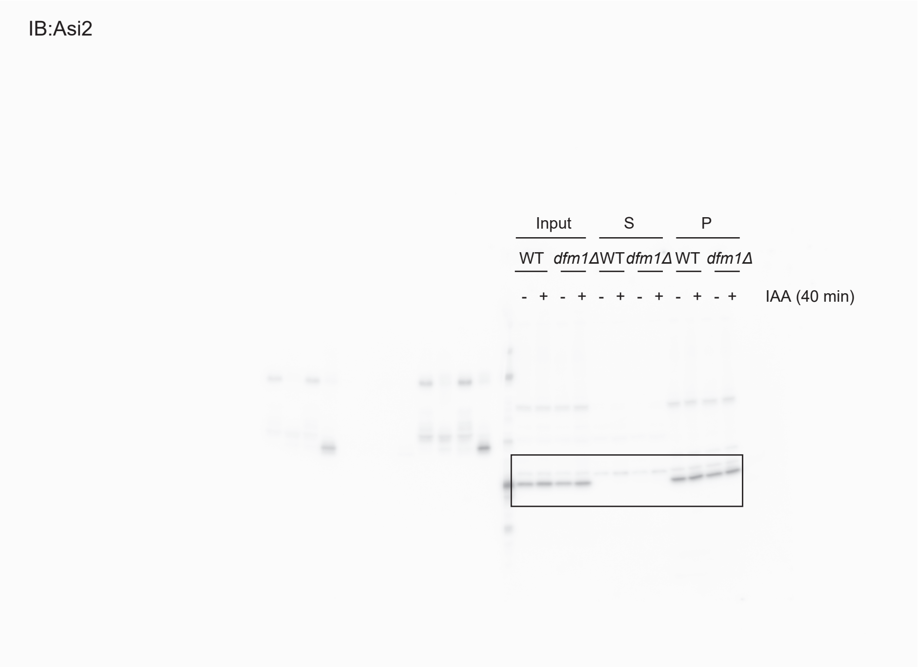
IB:Asi2
Input
S
P
WT
dfm1Δ
WT
dfm1Δ
WT
dfm1Δ
- + - + - + - + - + - +
IAA (40 min)

Fig. 5B

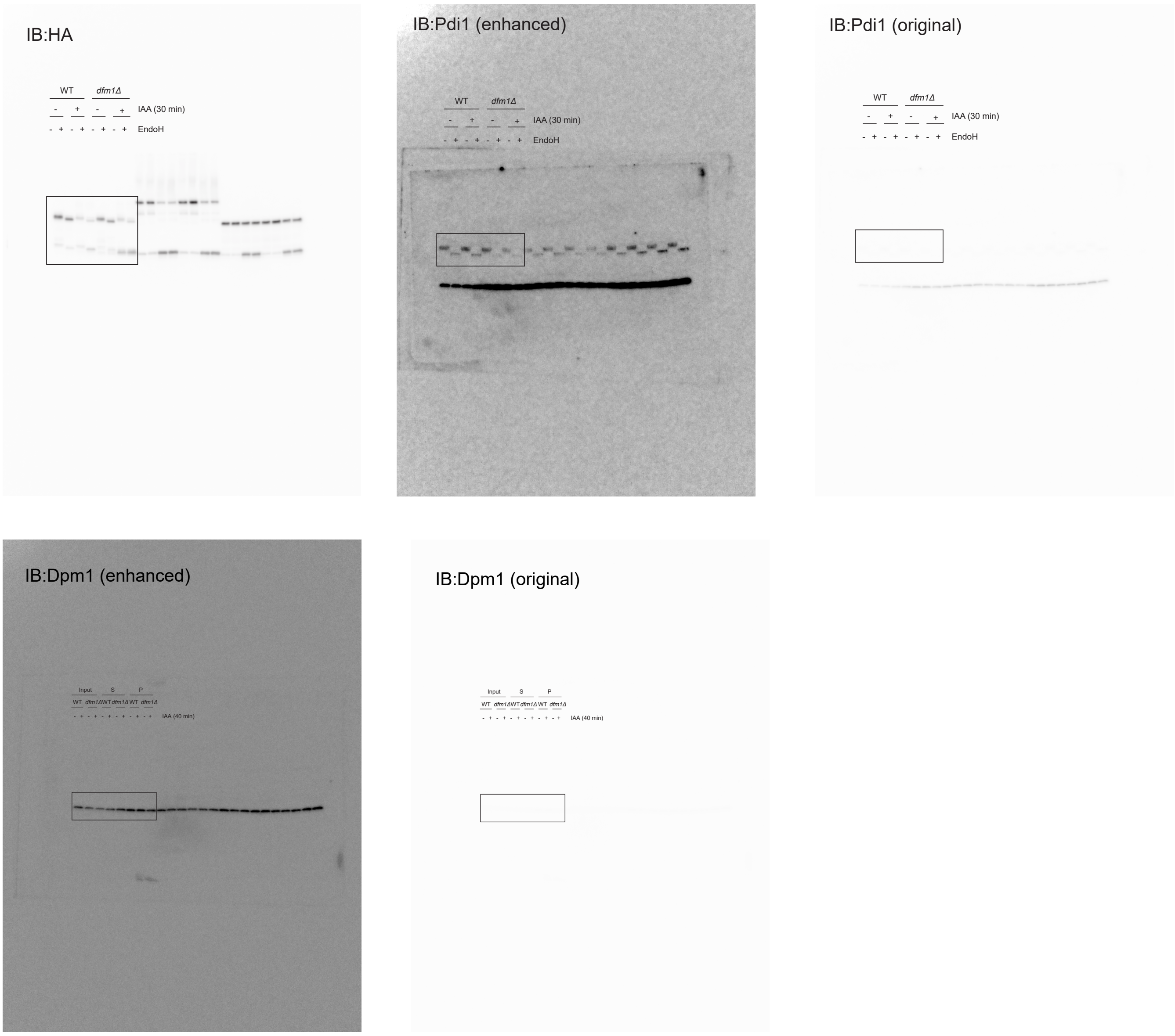
IB:HA
WT dfm1Δ
- + - + IAA (30 min)
- + - + - + - + EndoH
IB:Pdi1 (enhanced)
WT dfm1Δ
- + - + IAA (30 min)
- + - + - + - + EndoH
IB:Pdi1 (original)
WT dfm1Δ
- + - + IAA (30 min)
- + - + - + - + EndoH
IB:Dpm1 (enhanced)
Input S P
WT dfm1Δ WT dfm1Δ WT dfm1Δ
- + - + - + - + - + - + IAA (40 min)
IB:Dpm1 (original)
Input S P
WT dfm1Δ WT dfm1Δ WT dfm1Δ
- + - + - + - + - + - + IAA (40 min)

Fig. 5D

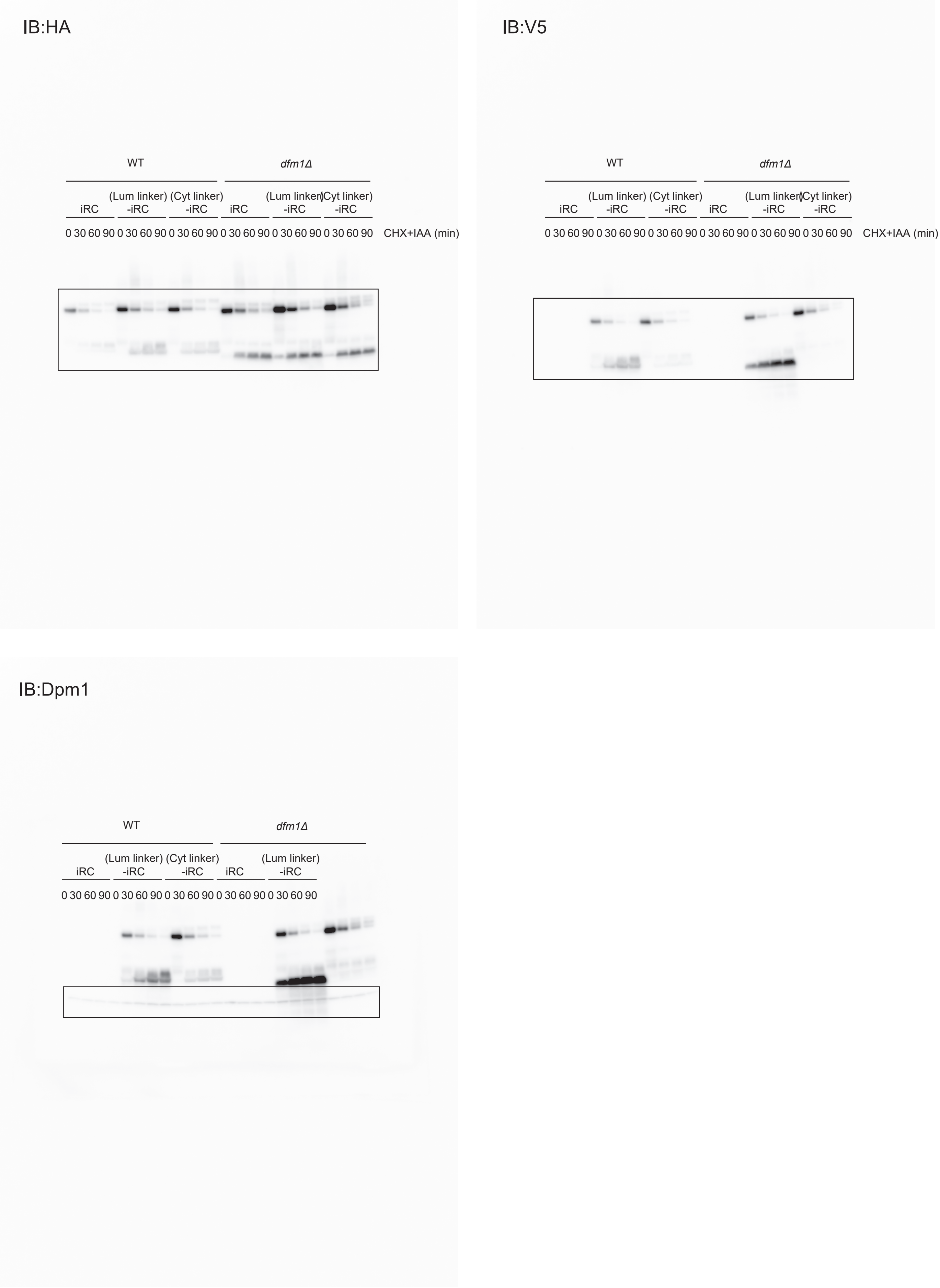
IB:HA
WT
dfm1Δ
iRC
(Lum linker) -iRC
(Cyt linker) -iRC
iRC
(Lum linker) -iRC
(Cyt linker) -iRC
0 30 60 90 0 30 60 90 0 30 60 90 0 30 60 90 0 30 60 90 0 30 60 90
CHX+IAA (min)
IB:V5
WT
dfm1Δ
iRC
(Lum linker) -iRC
(Cyt linker) -iRC
iRC
(Lum linker) -iRC
(Cyt linker) -iRC
0 30 60 90 0 30 60 90 0 30 60 90 0 30 60 90 0 30 60 90 0 30 60 90
CHX+IAA (min)
IB:Dpm1
WT
dfm1Δ
iRC
(Lum linker) -iRC
(Cyt linker) -iRC
iRC
(Lum linker) -iRC
0 30 60 90 0 30 60 90 0 30 60 90 0 30 60 90 0 30 60 90
