# Supplementary material for: The derlin Dfm1 couples retrotranslocation of a folded protein domain to its proteasomal degradation
Source: J Cell Biol. 2024 Mar 5;223(5):e202308074. doi: 10.1083/jcb.202308074 (PMC11066878; doi:10.1083/jcb.202308074)

Fig. 6A

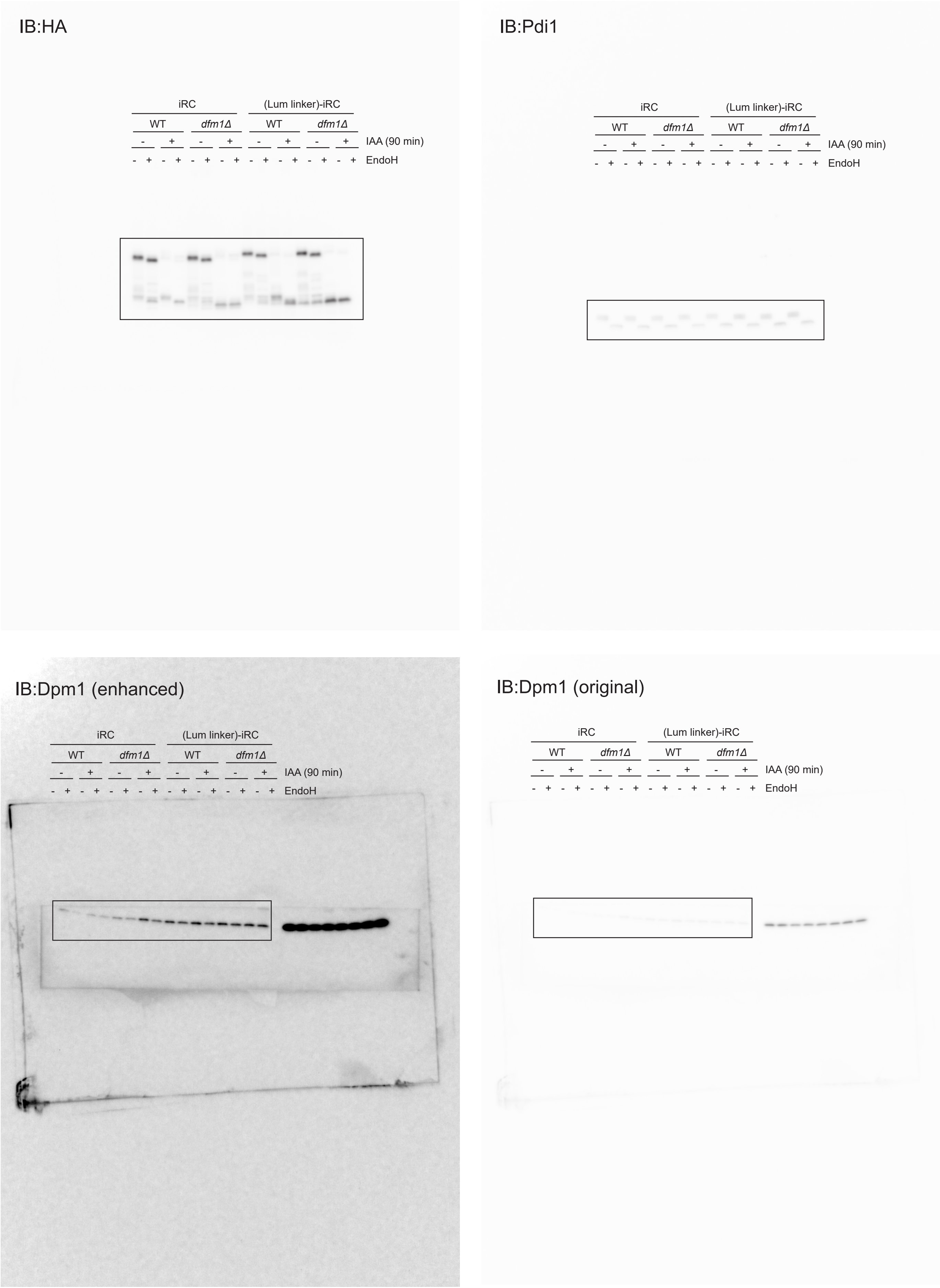

IB:HA
iRC
(Lum linker)-iRC
WT
dfm1Δ
WT
dfm1Δ
- + - + - + - +
IAA (90 min)
- + - + - + - + - + - + - + - +
EndoH
IB:Pdi1
iRC
(Lum linker)-iRC
WT
dfm1Δ
WT
dfm1Δ
- + - + - + - +
IAA (90 min)
- + - + - + - + - + - + - + - +
EndoH
IB:Dpm1 (enhanced)
iRC
(Lum linker)-iRC
WT
dfm1Δ
WT
dfm1Δ
- + - + - + - +
IAA (90 min)
- + - + - + - + - + - + - + - +
EndoH
IB:Dpm1 (original)
iRC
(Lum linker)-iRC
WT
dfm1Δ
WT
dfm1Δ
- + - + - + - +
IAA (90 min)
- + - + - + - + - + - + - + - +
EndoH

Fig. 6B

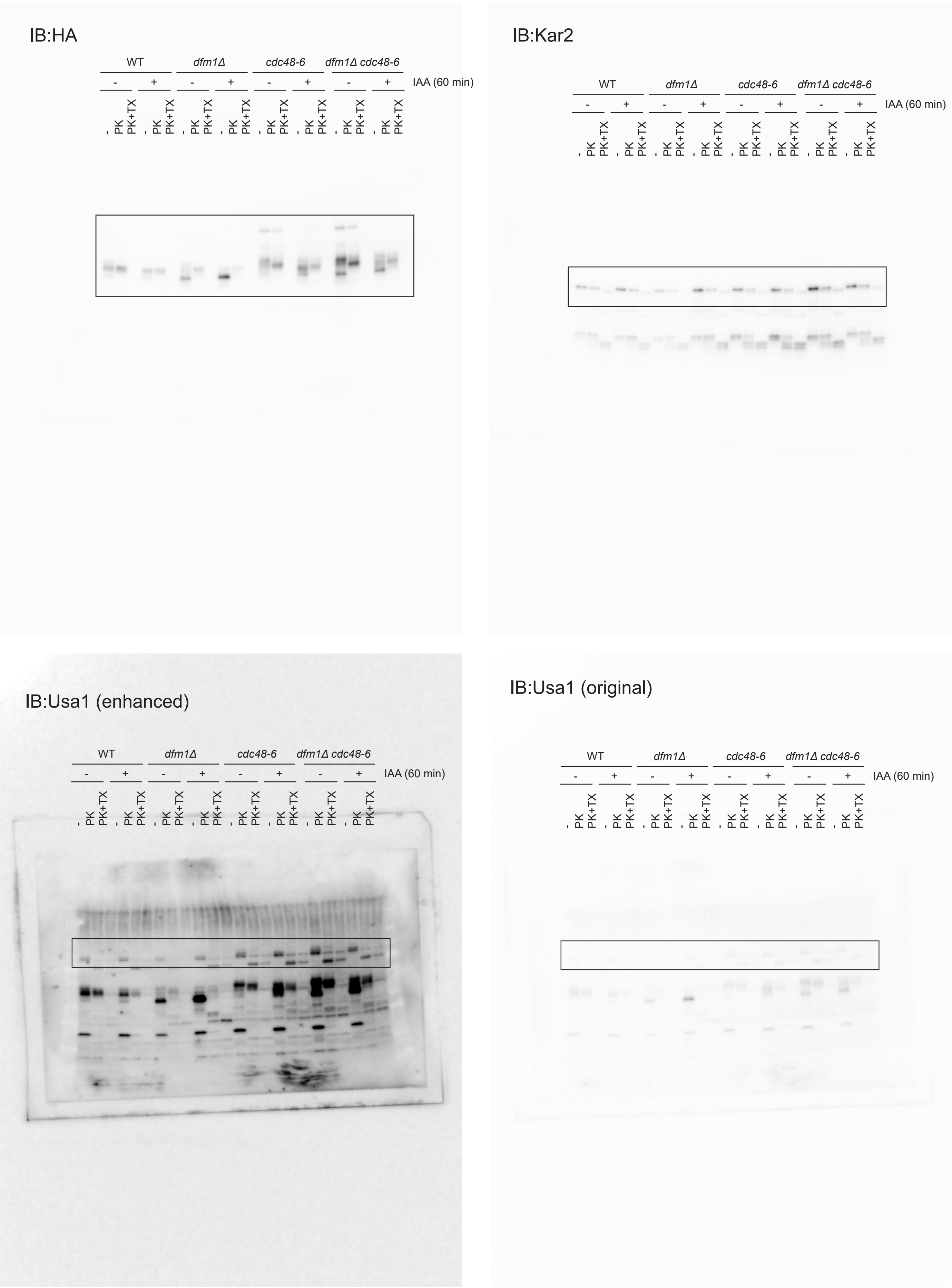
IB:HA
WT
dfm1Δ
cdc48-6
dfm1Δ cdc48-6
- + - + - + - +
IAA (60 min)
- PK PK+TX - PK PK+TX - PK PK+TX - PK PK+TX - PK PK+TX - PK PK+TX - PK PK+TX - PK PK+TX
IB:Kar2
WT
dfm1Δ
cdc48-6
dfm1Δ cdc48-6
- + - + - + - +
IAA (60 min)
- PK PK+TX - PK PK+TX - PK PK+TX - PK PK+TX - PK PK+TX - PK PK+TX - PK PK+TX - PK PK+TX
IB:Usa1 (enhanced)
WT
dfm1Δ
cdc48-6
dfm1Δ cdc48-6
- + - + - + - +
IAA (60 min)
- PK PK+TX - PK PK+TX - PK PK+TX - PK PK+TX - PK PK+TX - PK PK+TX - PK PK+TX - PK PK+TX
IB:Usa1 (original)
WT
dfm1Δ
cdc48-6
dfm1Δ cdc48-6
- + - + - + - +
IAA (60 min)
- PK PK+TX - PK PK+TX - PK PK+TX - PK PK+TX - PK PK+TX - PK PK+TX - PK PK+TX - PK PK+TX

Fig. 6C

IB:HA

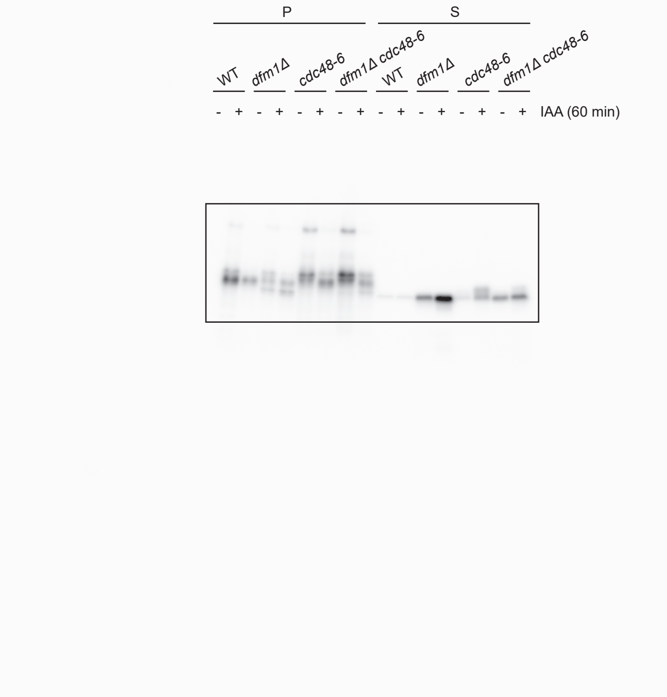

IB:Dpm1

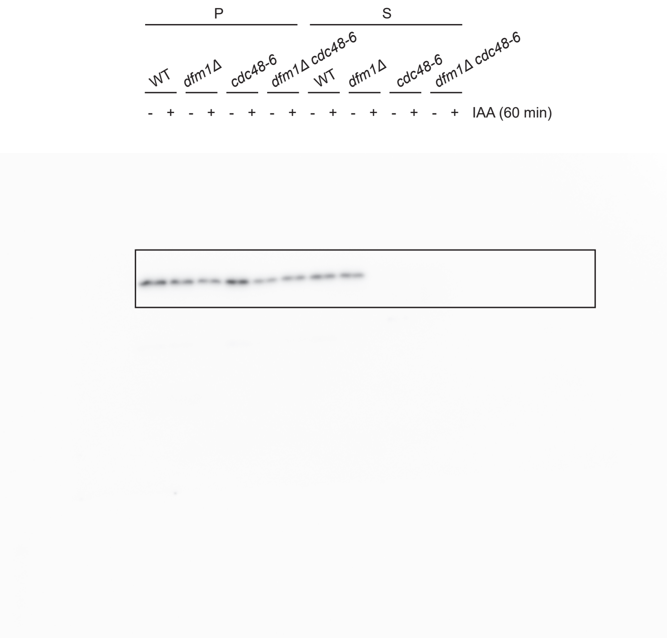

IB:Kar2

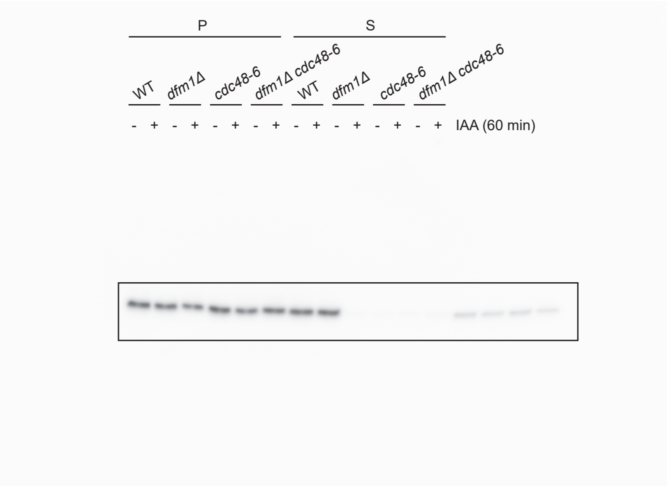

IB:Pgk1

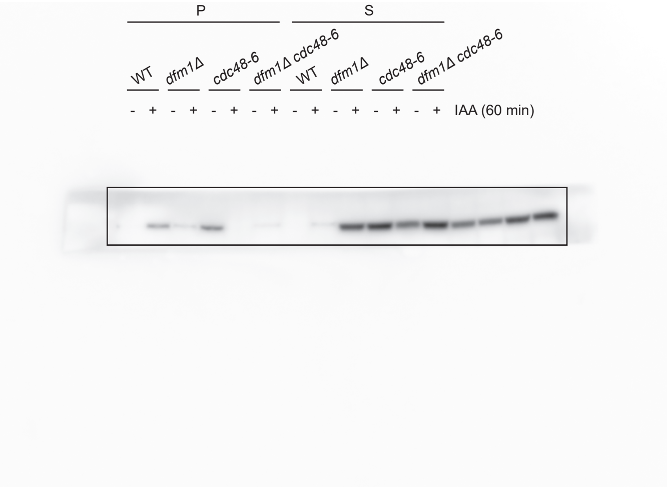

Supplement: SourceData F6 — is the source file for Fig. 6. [file JCB_202308074_SourceDataF6.pdf]
