# Supplementary material for: The derlin Dfm1 couples retrotranslocation of a folded protein domain to its proteasomal degradation
Source: J Cell Biol. 2024 Mar 5;223(5):e202308074. doi: 10.1083/jcb.202308074 (PMC11066878; doi:10.1083/jcb.202308074)

Fig. S1A

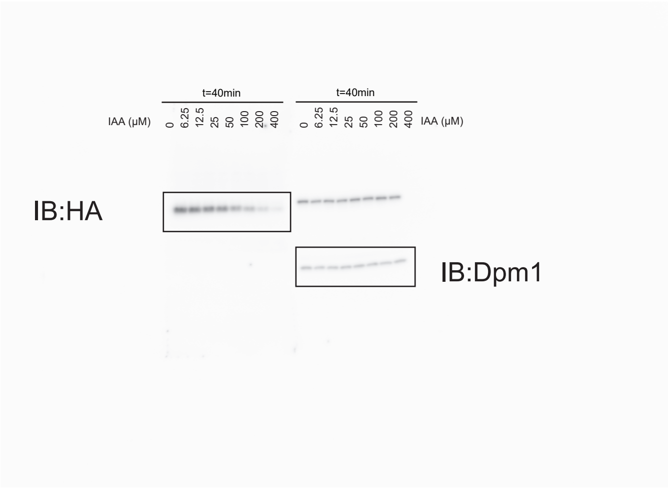
t=40min
t=40min
IAA (µM)
0
6.25
12.5
25
50
100
200
400
IAA (µM)
IB:HA
IB:Dpm1

Fig. S1B

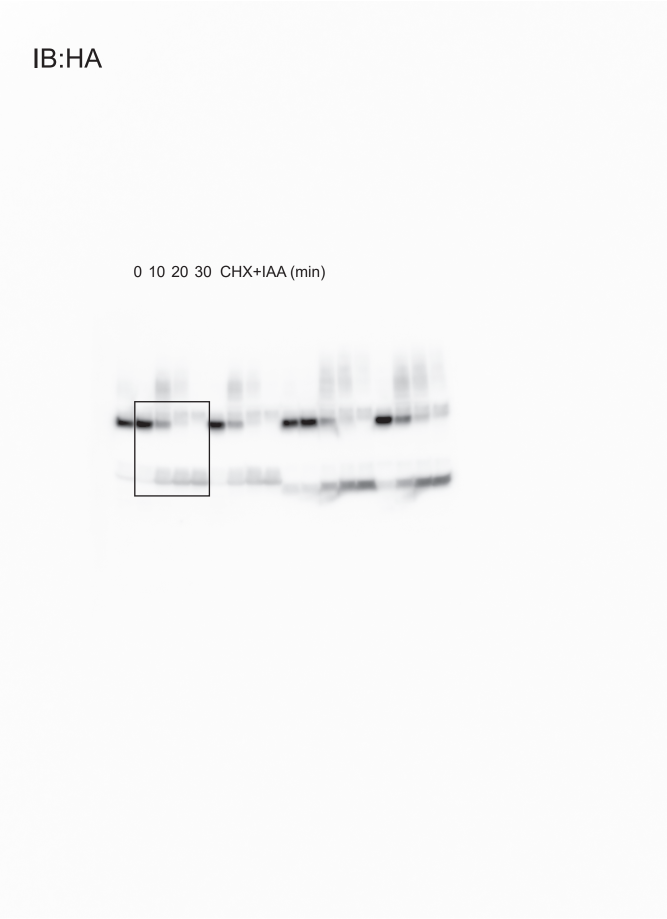
IB:HA
0 10 20 30 CHX+IAA (min)

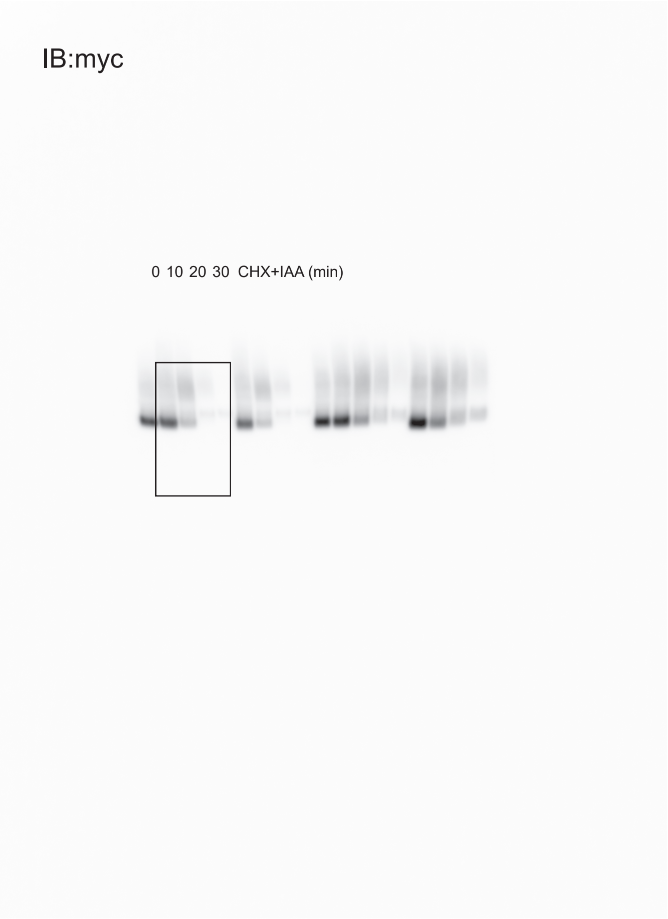
IB:myc
0 10 20 30 CHX+IAA (min)

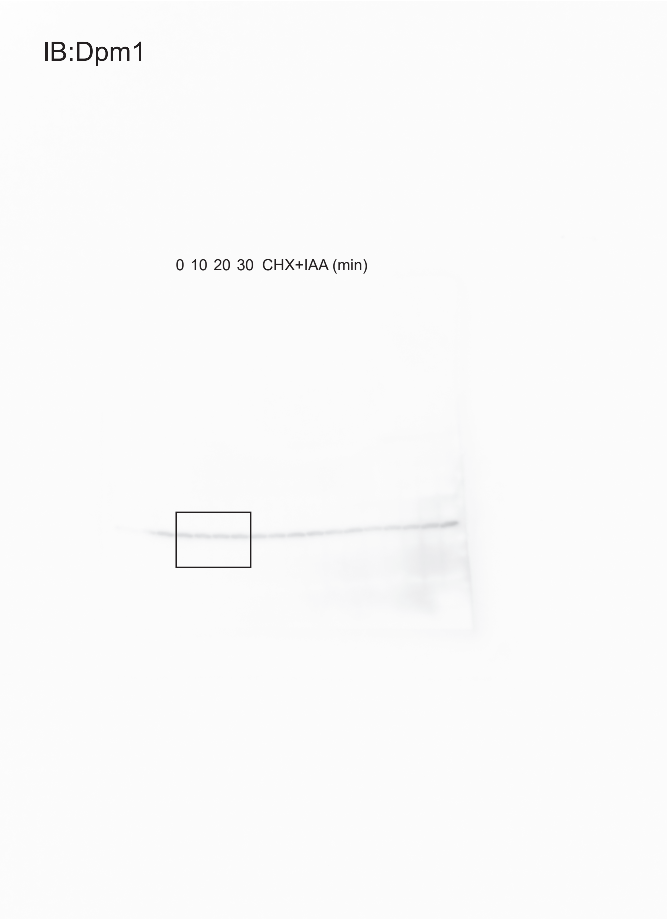
IB:Dpm1
0 10 20 30 CHX+IAA (min)

Fig. S1C

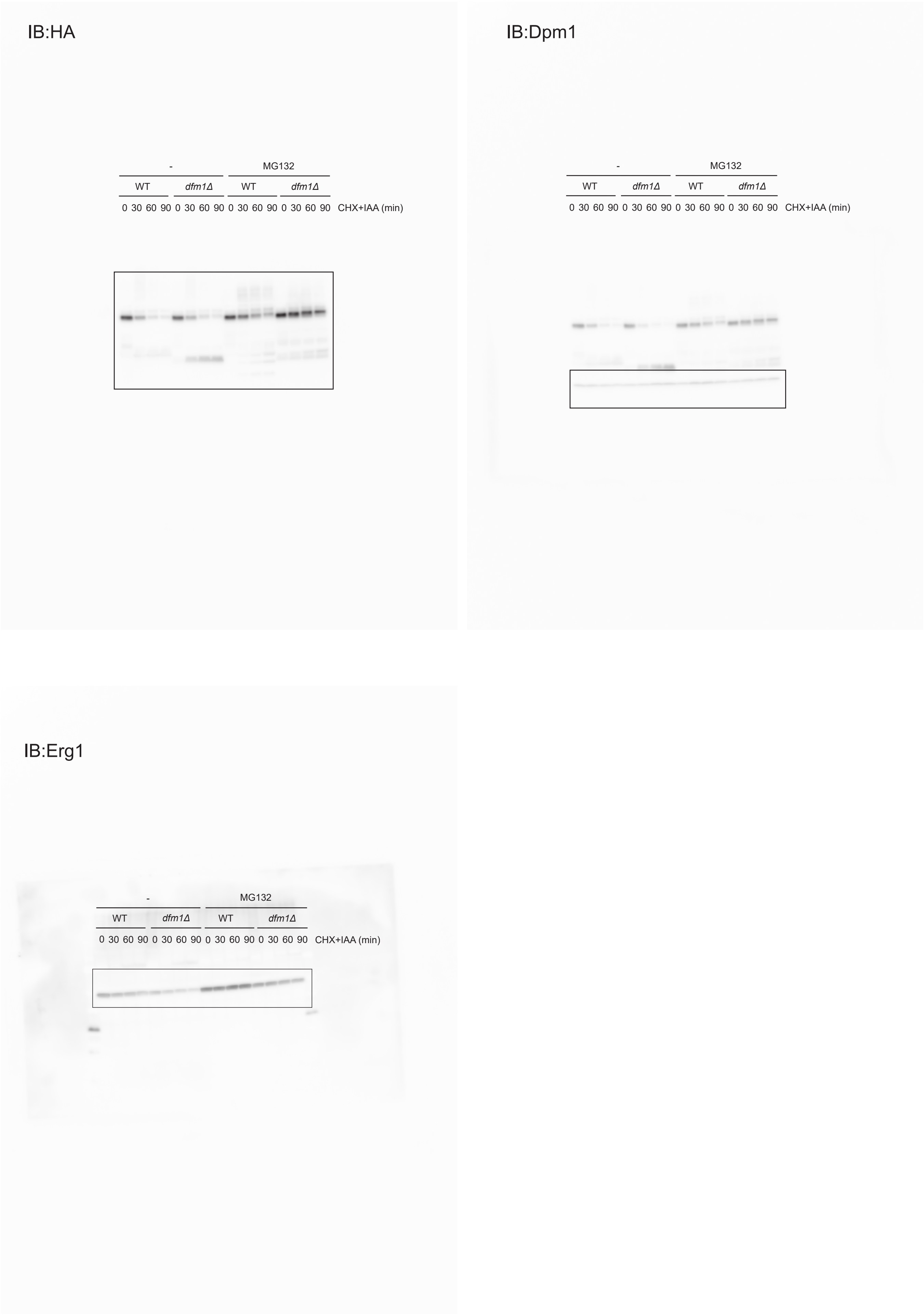
IB:HA
-
MG132
WT
dfm1Δ
WT
dfm1Δ
0 30 60 90 0 30 60 90 0 30 60 90 0 30 60 90 CHX+IAA (min)
IB:Dpm1
-
MG132
WT
dfm1Δ
WT
dfm1Δ
0 30 60 90 0 30 60 90 0 30 60 90 0 30 60 90 CHX+IAA (min)
IB:Erg1
-
MG132
WT
dfm1Δ
WT
dfm1Δ
0 30 60 90 0 30 60 90 0 30 60 90 0 30 60 90 CHX+IAA (min)

Fig. S1D

IB:HA

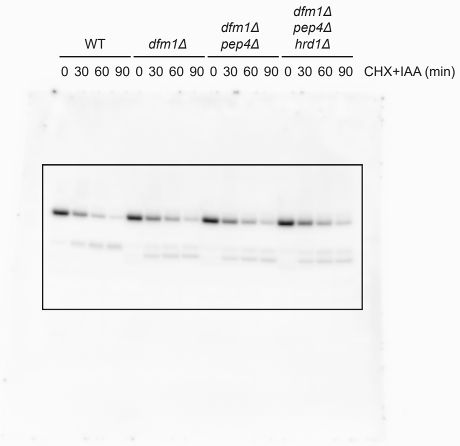

IB:Dpm1

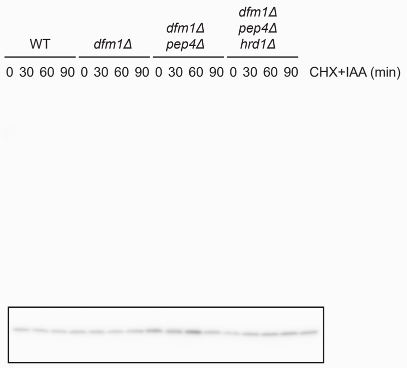

Supplement: SourceData FS1 — is the source file for Fig. S1. [file JCB_202308074_SourceDataFS1.pdf]
