# Supplementary material for: The derlin Dfm1 couples retrotranslocation of a folded protein domain to its proteasomal degradation
Source: J Cell Biol. 2024 Mar 5;223(5):e202308074. doi: 10.1083/jcb.202308074 (PMC11066878; doi:10.1083/jcb.202308074)
Supplement: SourceData FS4 — is the source file for Fig. S4. [file JCB_202308074_SourceDataFS4.pdf]

Fig. S4A

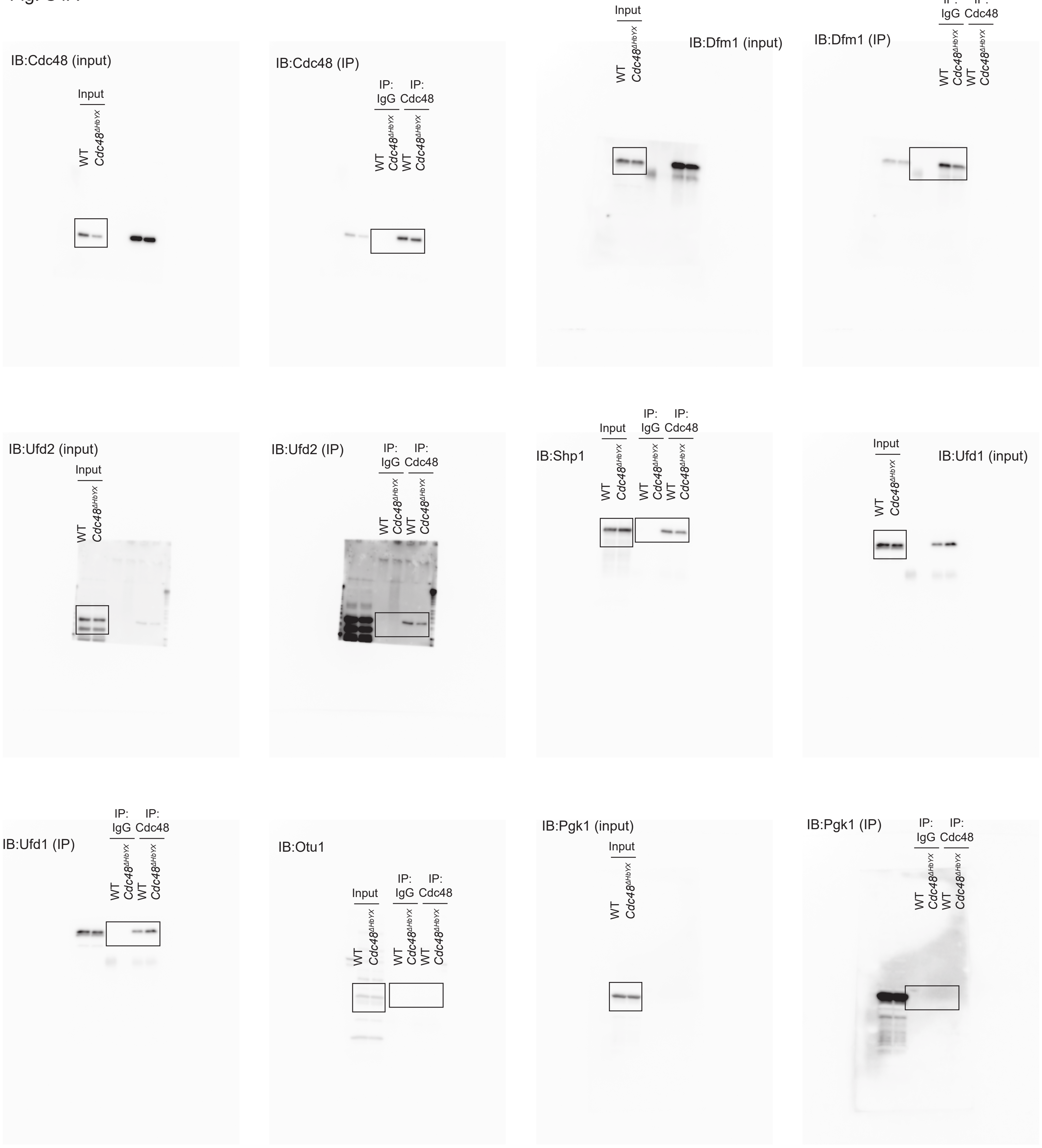
IB:Cdc48 (input)
Input
WT
Cdc48$^{\Delta HbYX}$
IB:Cdc48 (IP)
IP: IgG
IP: Cdc48
WT
Cdc48$^{\Delta HbYX}$
WT
Cdc48$^{\Delta HbYX}$
IB:Dfm1 (input)
Input
WT
Cdc48$^{\Delta HbYX}$
IB:Dfm1 (IP)
IP: IgG
IP: Cdc48
WT
Cdc48$^{\Delta HbYX}$
WT
Cdc48$^{\Delta HbYX}$
IB:Ufd2 (input)
Input
WT
Cdc48$^{\Delta HbYX}$
IB:Ufd2 (IP)
IP: IgG
IP: Cdc48
WT
Cdc48$^{\Delta HbYX}$
WT
Cdc48$^{\Delta HbYX}$
IB:Shp1
Input
IP: IgG
IP: Cdc48
WT
Cdc48$^{\Delta HbYX}$
WT
Cdc48$^{\Delta HbYX}$
WT
Cdc48$^{\Delta HbYX}$
IB:Ufd1 (input)
Input
WT
Cdc48$^{\Delta HbYX}$
IB:Ufd1 (IP)
IP: IgG
IP: Cdc48
WT
Cdc48$^{\Delta HbYX}$
WT
Cdc48$^{\Delta HbYX}$
IB:Otu1
Input
IP: IgG
IP: Cdc48
WT
Cdc48$^{\Delta HbYX}$
WT
Cdc48$^{\Delta HbYX}$
WT
Cdc48$^{\Delta HbYX}$
IB:Pgk1 (input)
Input
WT
Cdc48$^{\Delta HbYX}$
IB:Pgk1 (IP)
IP: IgG
IP: Cdc48
WT
Cdc48$^{\Delta HbYX}$
WT
Cdc48$^{\Delta HbYX}$

Fig. S4B

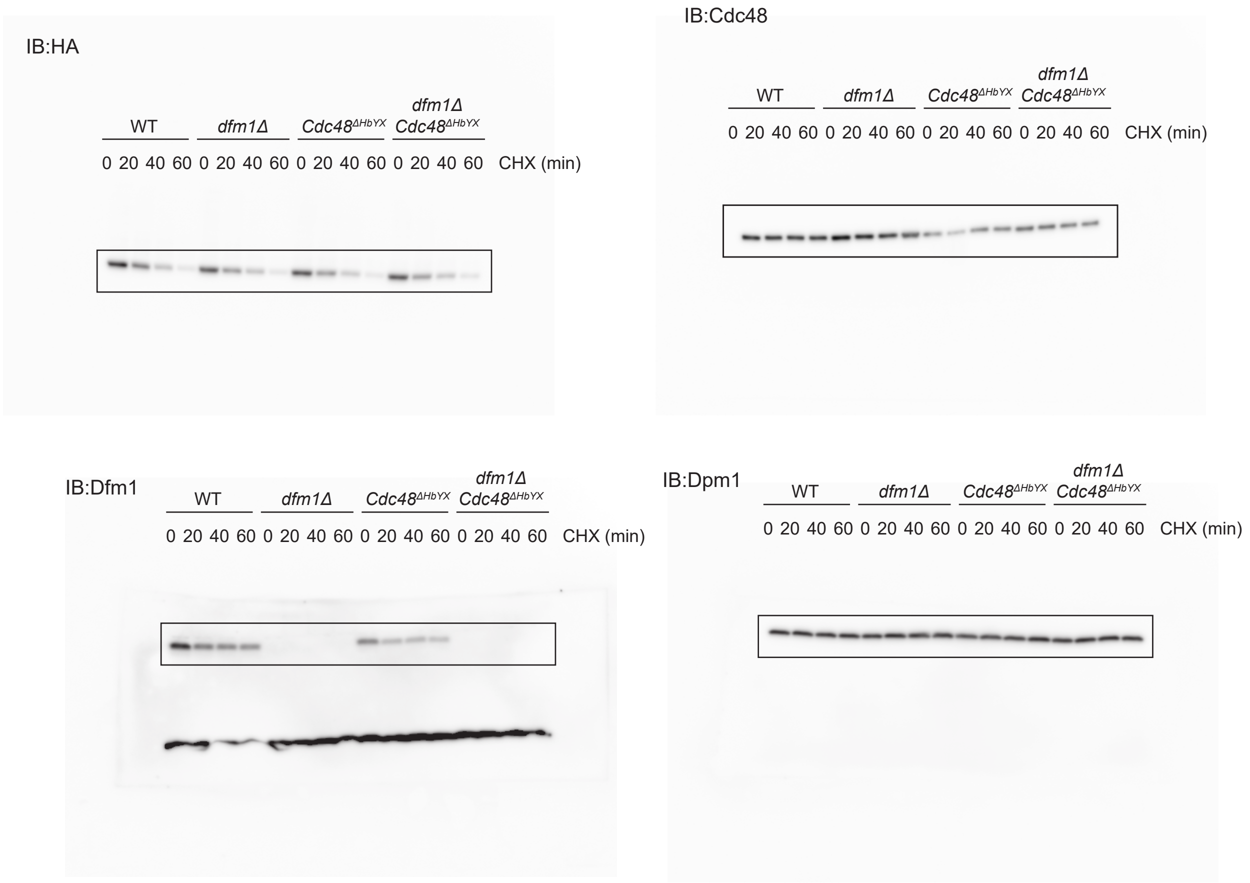
IB:HA
WT
dfm1Δ
$Cdc48^{\Delta HbYX}$
dfm1Δ $Cdc48^{\Delta HbYX}$
0 20 40 60 0 20 40 60 0 20 40 60 0 20 40 60 CHX (min)
IB:Cdc48
WT
dfm1Δ
$Cdc48^{\Delta HbYX}$
dfm1Δ $Cdc48^{\Delta HbYX}$
0 20 40 60 0 20 40 60 0 20 40 60 0 20 40 60 CHX (min)
IB:Dfm1
WT
dfm1Δ
$Cdc48^{\Delta HbYX}$
dfm1Δ $Cdc48^{\Delta HbYX}$
0 20 40 60 0 20 40 60 0 20 40 60 0 20 40 60 CHX (min)
IB:Dpm1
WT
dfm1Δ
$Cdc48^{\Delta HbYX}$
dfm1Δ $Cdc48^{\Delta HbYX}$
0 20 40 60 0 20 40 60 0 20 40 60 0 20 40 60 CHX (min)
