# Supplementary material for: The derlin Dfm1 couples retrotranslocation of a folded protein domain to its proteasomal degradation
Source: J Cell Biol. 2024 Mar 5;223(5):e202308074. doi: 10.1083/jcb.202308074 (PMC11066878; doi:10.1083/jcb.202308074)
Supplement: SourceData FS5 — is the source file for Fig. S5. [file JCB_202308074_SourceDataFS5.pdf]

Fig. S5A

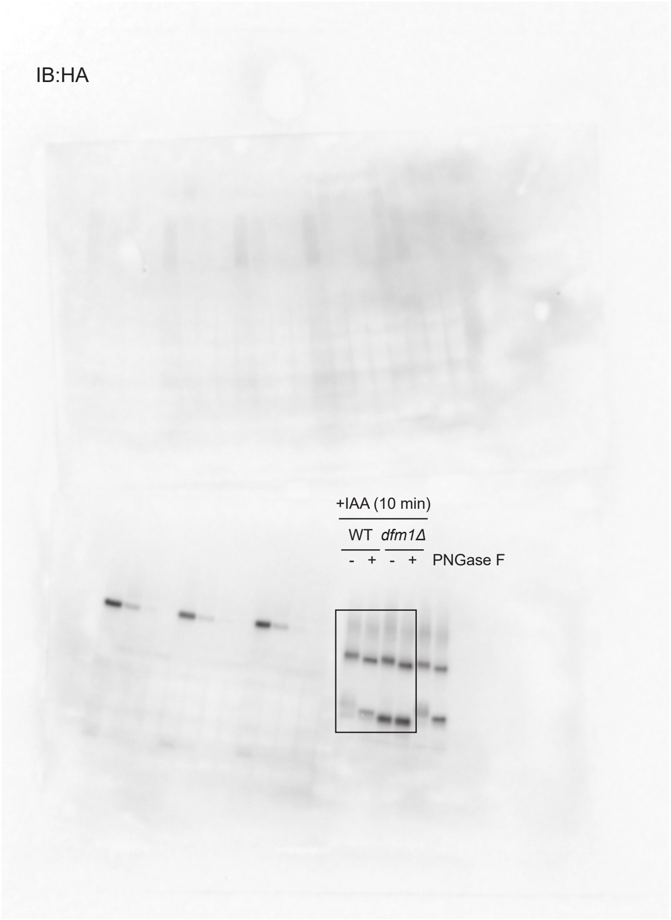

Fig. S5B

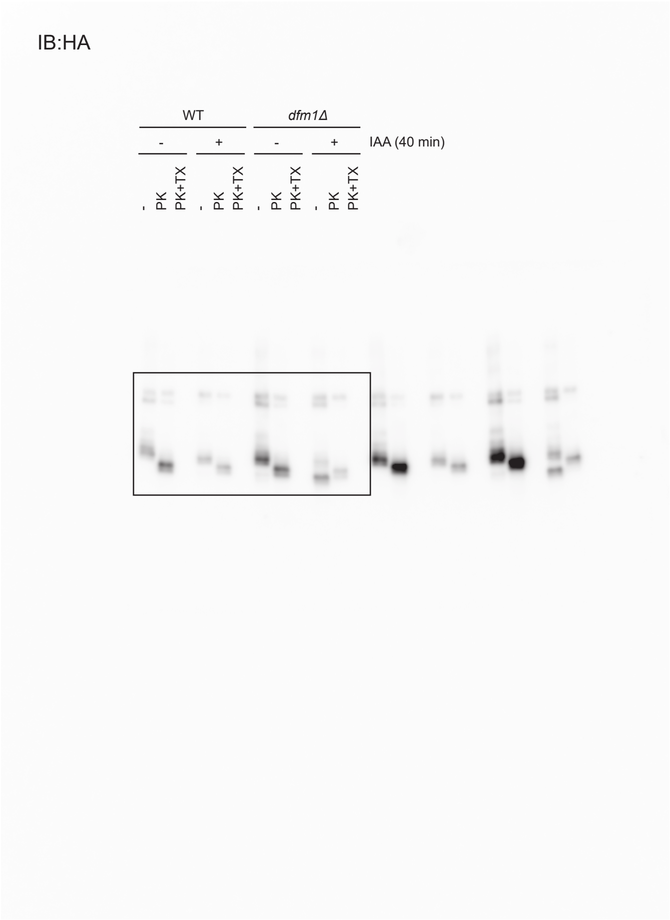

IB:HA
WT
dfm1Δ
-
+
-
+
IAA (40 min)
- PK PK+TX - PK PK+TX - PK PK+TX - PK PK+TX

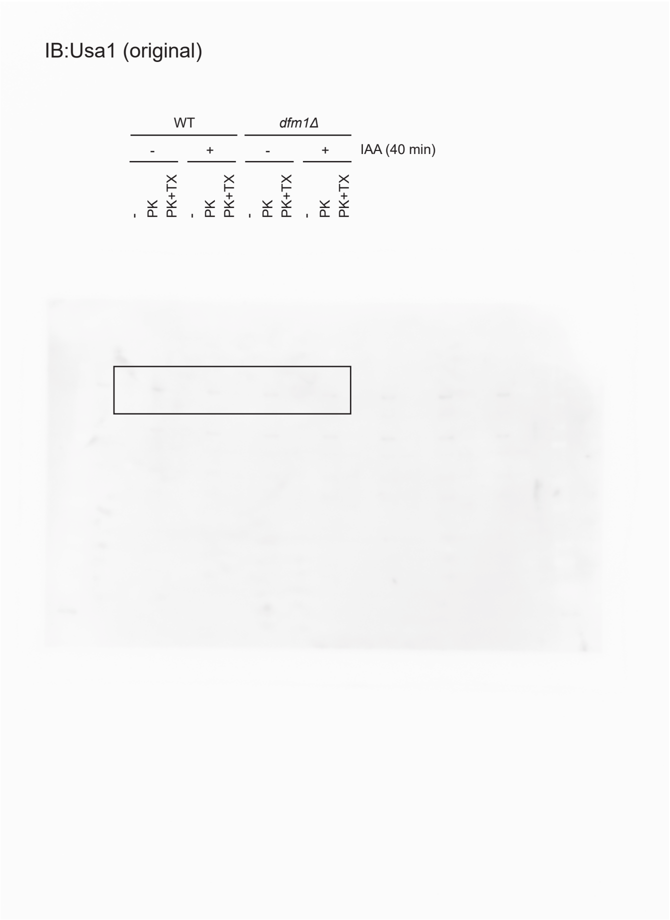

IB:Usa1 (original)
WT
dfm1Δ
-
+
-
+
IAA (40 min)
- PK PK+TX - PK PK+TX - PK PK+TX - PK PK+TX

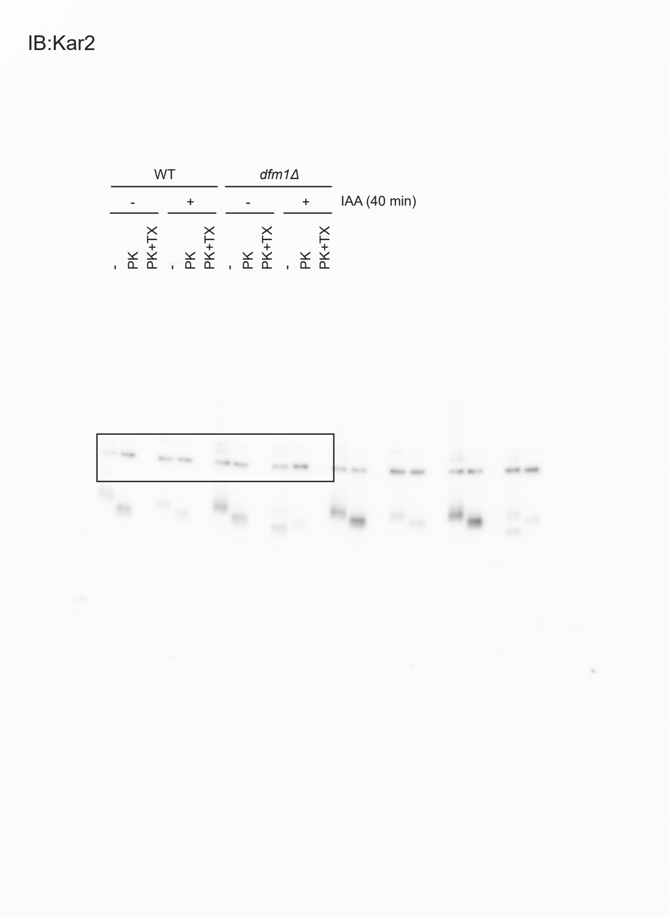

IB:Kar2
WT
dfm1Δ
-
+
-
+
IAA (40 min)
- PK PK+TX - PK PK+TX - PK PK+TX - PK PK+TX

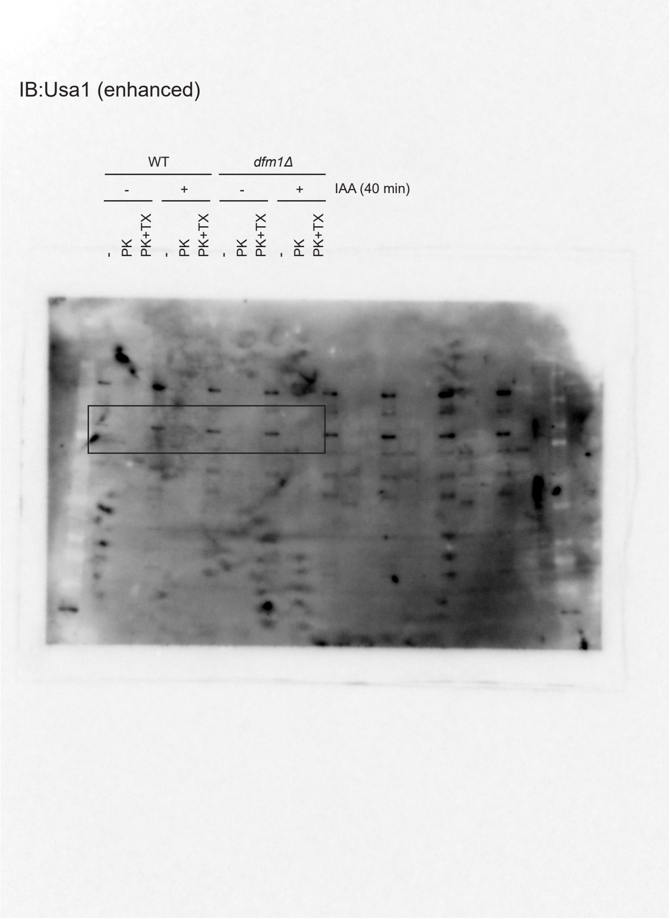

IB:Usa1 (enhanced)
WT
dfm1Δ
-
+
-
+
IAA (40 min)
- PK PK+TX - PK PK+TX - PK PK+TX - PK PK+TX

Fig. S5C

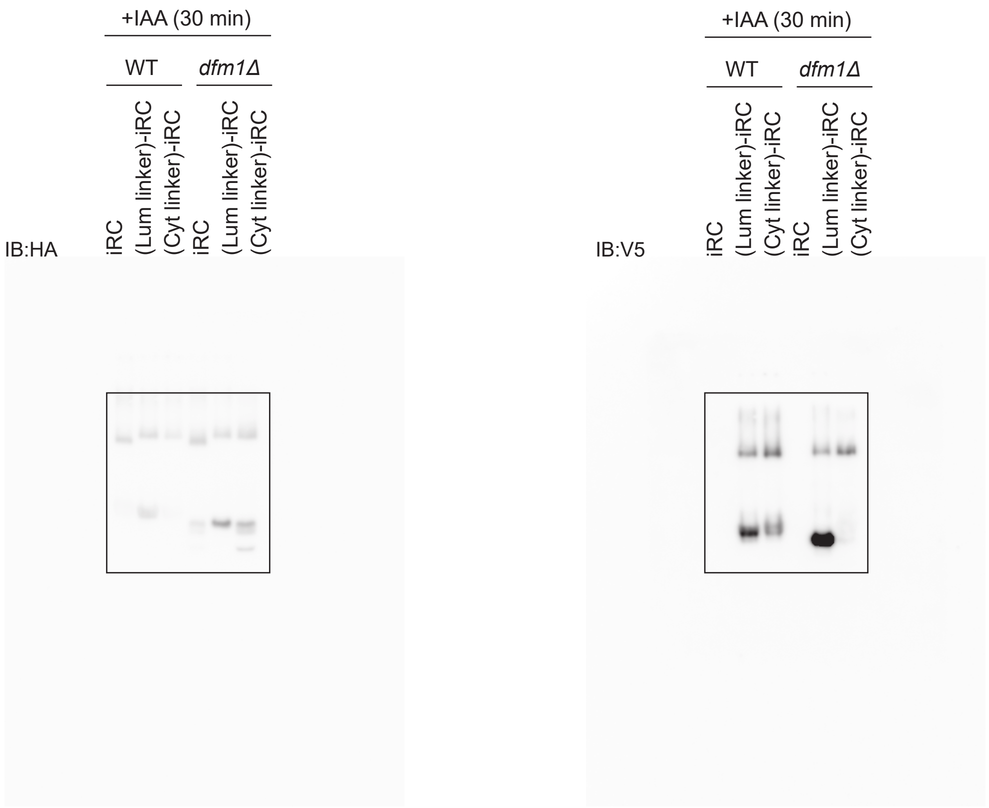
+IAA (30 min)
WT
dfm1Δ
iRC
(Lum linker)-iRC
(Cyt linker)-iRC
iRC
(Lum linker)-iRC
(Cyt linker)-iRC
IB:HA
+IAA (30 min)
WT
dfm1Δ
iRC
(Lum linker)-iRC
(Cyt linker)-iRC
iRC
(Lum linker)-iRC
(Cyt linker)-iRC
IB:V5

Fig. S5D

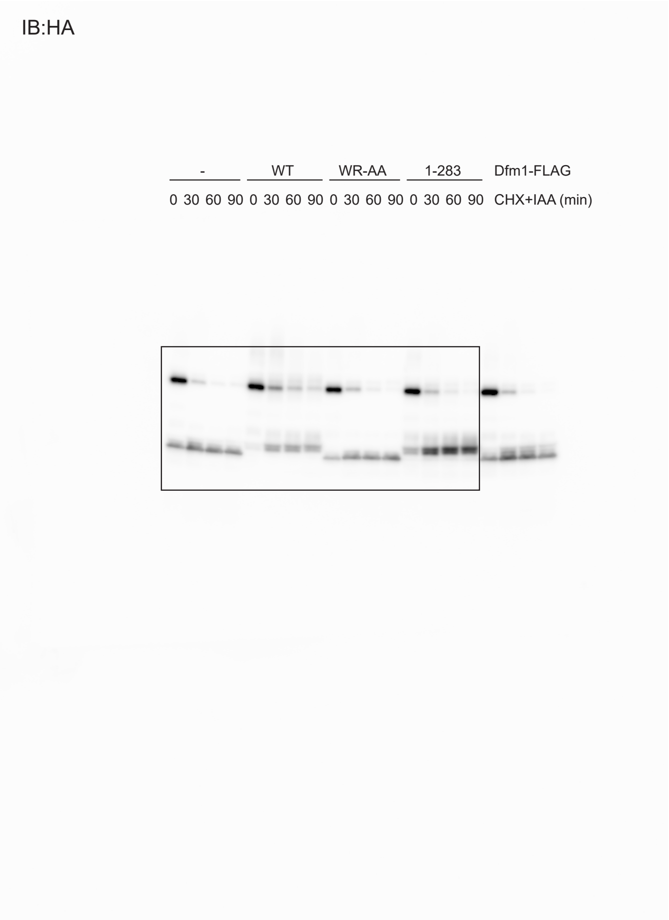
IB:HA
- WT WR-AA 1-283 Dfm1-FLAG
0 30 60 90 0 30 60 90 0 30 60 90 0 30 60 90 CHX+IAA (min)

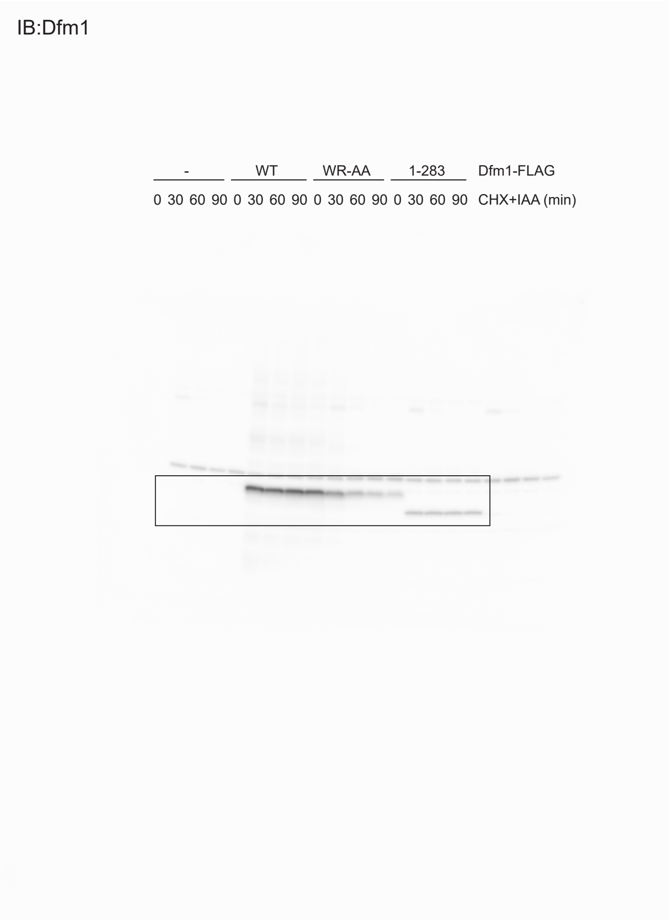
IB:Dfm1
- WT WR-AA 1-283 Dfm1-FLAG
0 30 60 90 0 30 60 90 0 30 60 90 0 30 60 90 CHX+IAA (min)

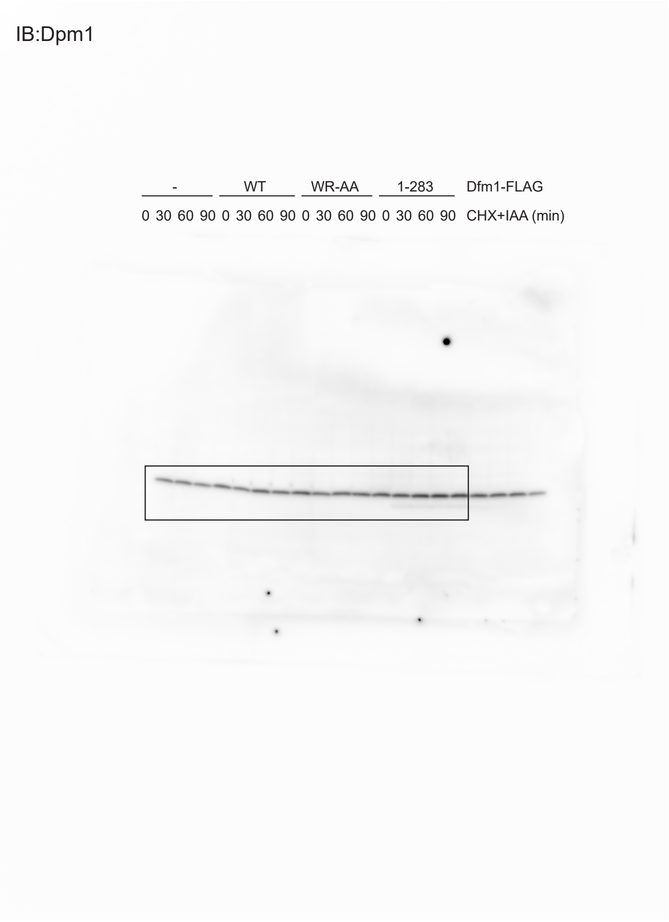
IB:Dpm1
- WT WR-AA 1-283 Dfm1-FLAG
0 30 60 90 0 30 60 90 0 30 60 90 0 30 60 90 CHX+IAA (min)

Fig. S5E

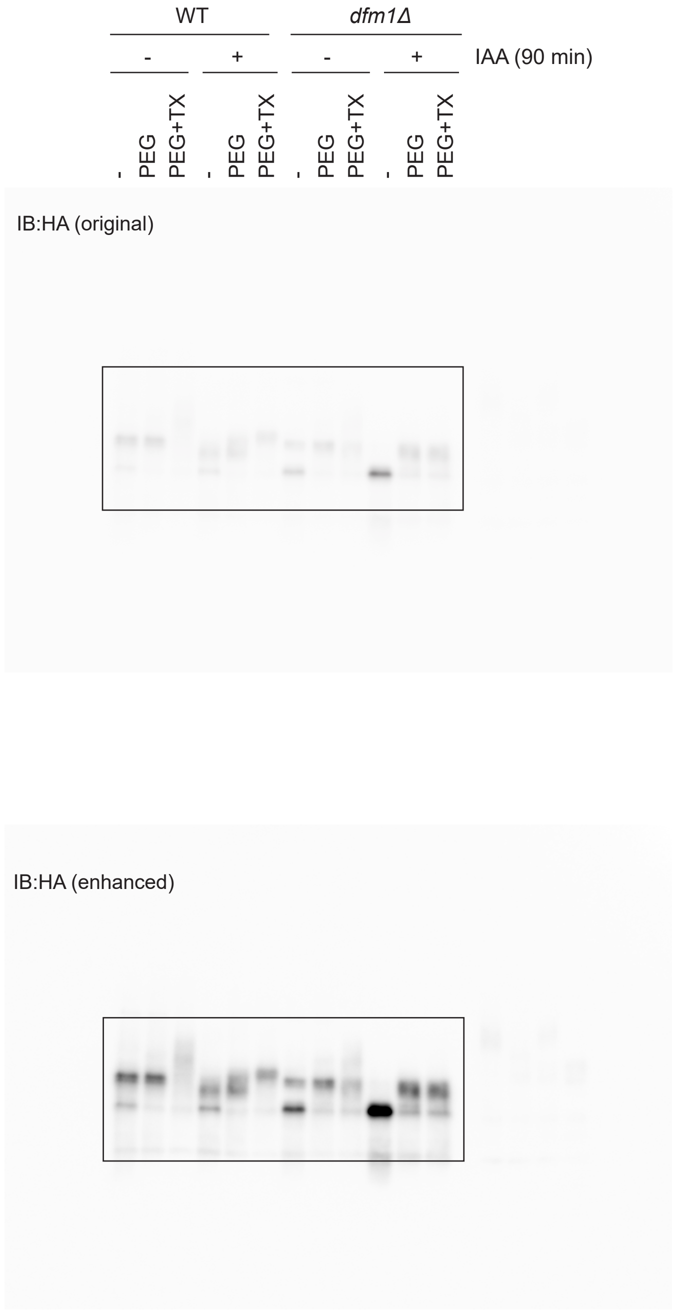
WT
dfm1Δ
-
+
-
+
IAA (90 min)
-
PEG
PEG+TX
-
PEG
PEG+TX
-
PEG
PEG+TX
-
PEG
PEG+TX
IB:HA (original)
IB:HA (enhanced)
